# The Impact of Growth Years on the Medicinal Material Characteristics and Metabolites of *Stellaria dichotoma* L. var. *lanceolata* Bge. Reveals the Optimal Harvest Age

**DOI:** 10.3390/plants12122286

**Published:** 2023-06-12

**Authors:** Zhenkai Li, Lu Feng, Hong Wang, Lin Zhang, Haishan Li, Yanqing Li, Pilian Niu, Gege Tian, Yan Yang, Xiangui Mei, Li Peng

**Affiliations:** 1 School of Life Sciences, Ningxia University, Yinchuan 750021, China; 12020140069@stu.nxu.edu.cn (Z.L.); tiangege0212@163.com (G.T.);; 2 State Key Laboratory of Crop Biology, College of Agronomy, Shandong Agricultural University, Taian 271018, China; 3 Ningxia Natural Medicine Engineering Technology Research Center, Yinchuan 750021, China

**Keywords:** *Stellaria dichotoma* L. var. *lanceolata* Bge., growth years, metabolomics, medicinal materials characteristic, components accumulation, quality formation

## Abstract

The original plant of Chinese medicine Stellariae Radix (Yin Chai Hu) is *Stellaria dichotoma* L. var. *lanceolata* Bge (abbreviated as SDL). SDL is a perennial herbaceous plant and a characteristic crop in Ningxia. Growth years are vital factors that affect the quality of perennial medicinal materials. This study aims to investigate the impact of growth years on SDL and screen for the optimal harvest age by comparing the medicinal material characteristics of SDL with different growth years. Additionally, metabolomics analysis using UHPLC-Q-TOF MS was employed to investigate the impact of growth years on the accumulation of metabolites in SDL. The results show that the characteristics of medicinal materials and the drying rate of SDL gradually increase with the increase in growth years. The fastest development period of SDL occurred during the first 3 years, after which the development slowed down. Medicinal materials characteristics of 3-year-old SDL exhibited mature qualities with a high drying rate, methanol extract content, and the highest content of total sterols and total flavonoids. A total of 1586 metabolites were identified, which were classified into 13 major classes with more than 50 sub-classes. Multivariate statistical analysis indicated significant differences in the diversity of metabolites of SDL in different growth years, with greater differences observed in metabolites as the growth years increased. Moreover, different highly expressed metabolites in SDL at different growth years were observed: 1–2 years old was beneficial to the accumulation of more lipids, while 3–5 years old was conducive to accumulating more alkaloids, benzenoids, etc. Furthermore, 12 metabolites accumulating with growth years and 20 metabolites decreasing with growth years were screened, and 17 significantly different metabolites were noted in 3-year-old SDL. In conclusion, growth years not only influenced medicinal material characteristics, drying rate, content of methanol extract, and total sterol and flavonoid contents, but also had a considerable effect on SDL metabolites and metabolic pathways. SDL planted for 3 years presented the optimum harvest time. The screened significantly different metabolites with biological activity, such as rutin, cucurbitacin e, isorhamnetin-3-o-glucoside, etc., can be utilized as potential quality markers of SDL. This research provides references for studying the growth and development of SDL medicinal materials, the accumulation of metabolites, and the selection of optimal harvest time.

## 1. Introduction

Stellariae Radix (Yin Chai Hu) is a kind of Chinese herbal medicine that is commonly used in the treatment of yin-deficiency fever, infantile malnutrition fever, and other symptoms in traditional medicine, and it has good anti-inflammatory, anti-allergic, and anti-cancer effects [1]. *Stellaria dichotoma* L. var. *lanceolata* Bge. (abbreviated as SDL) is the original plant of Stellariae Radix, and its dried roots are used in medicine [2]. SDL is a perennial herbaceous plant and an important crop in Ningxia. In the 1980s, the province initiated the introduction and domestication of SDL, which has been under cultivation for over four decades. Owing to its exceptional drought and barren resistance, SDL has found widespread cultivation in harsh environments and emerged as a significant economic source for local farmers. At present, Tongxin County in Ningxia has become the largest supply area for SDL.

Growth years is an important factor affecting the quality and yield of herbs, especially for perennial root herbs [3,4]. A large body of research has demonstrated that extending the growth period for a certain period of time can enhance the yield of herbal medicine and aid in the accumulation of active compounds [5]. However, in planting production, most herbs only mature to a particular growth age, at which point the amount of active ingredients in medicinal plants reaches a maximum or gradually increases and their rate of growth and development begins to reduce [6]. For instance, *Morinda officinalis* reaches its peak level of activity at around 3 to 4 years of age [7]; when *Polygonatum* grows up to four years old, its polysaccharide content reaches the highest level, which is negatively correlated with the number of fibrous roots, leaf length, leaf width, stem diameter, and fresh weight of the shoot [8]; 1-year-old *licorice* has the highest levels of glycyrrhizin, 3-year-old *licorice* has the highest levels of neoisoliquiritin, and 4-year-old *licorice* has the highest levels of glycyrrhetinic acid, celery glycyrrhizin, glycyrrhizin, and celery isoglycyrrhi [9]. In addition, the accumulation of secondary metabolites in medicinal plants functions as a vital basis for controlling the quality of herbal medicines and maintaining their clinical effectiveness [10,11]. Growth years greatly affects secondary metabolites in medicinal plants, which has been confirmed in a variety of medicinal plants, such as *Bupleurum* [12], *Achyranthes bidentata* [13], and *Salvia miltiorrhiza* [14].

In the cultivation of SDL, it is generally believed that SDL planted for more than three years qualifies as a medicinal herb. Moreover, it is believed that the longer SDL is cultivated and the thicker its roots, the better its quality. However, this assertion lacks valid scientific evidence, particularly with regard to the impact on SDL metabolites, for which there are no scientific reports, at present. In this context, the research compares the characteristics and development of SDL medicinal materials with different growth years, detects the metabolites by ultra-high-performance liquid chromatography-tandem time of flight mass spectrometry (UHPLC-Q-TOF MS), and analyzes the effect of different growth years on the metabolites of SDL by using multidimensional statistical methods, aiming to provide a more scientific theoretical foundation for choosing the ideal harvesting year and producing SDL with high-yield and high-quality standards.

## 2. Results

### 2.1. Effect of Growth Years on Medicinal Materials Characteristics of SDL

**Figure 1:**
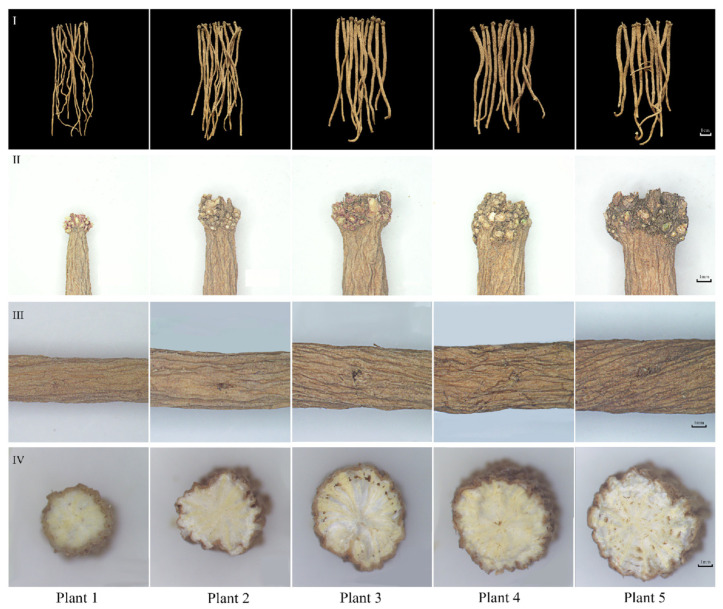
Characteristics of SDL medicinal materials with different growth years. (**I**) Full view of roots; (**II**) “Zhenzhupan”; (**III**) “Shayan”; (**IV**) yellow–white cross-sections.

**Figure 2:**
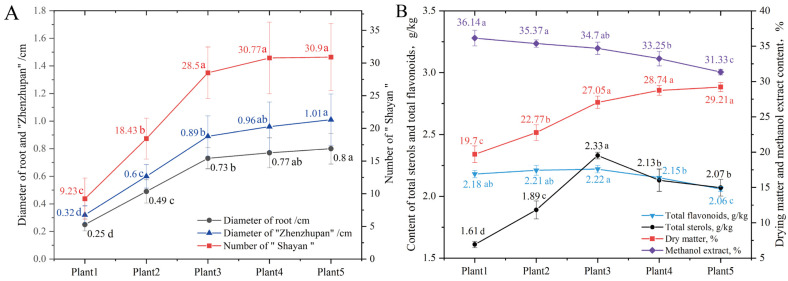
Changes in characteristics and components of SDL medicinal materials with growth years. (**A**) The variation trend of the diameter of the root head, diameter of “Zhenzhupan”, and the number of “Shayan”; (**B**) variation trend of the drying rate, methanol extract, content of total sterols and total flavonoids.The lowercase letters in the figure indicate the significant difference between the samples (*p* < 0.05), the same letter represents no significant difference, and different letters represent significant difference.

The results show that SDL with different growth years are characterized by “Zhenzhupan” (verrucous protuberances, residue of stem or rhizome), “Shayan” (pore-like or disc-shaped depressions), and yellow–white cross-sections, which are all main herbal characteristics of SDL according to the “Pharmacopoeia of the People’s Republic of China”. The differences were that the color of the herb became darker, the diameter of “Zhenzhupan” and the root head increased, the number of “Shayan” increased, and the yellow–white cross-sections became clearer as the growth years increased (Figure 1 and Figure 2A). In particular, from growth years 1 to 3, with the increase in growth years, the number of “Zhenzhupan” and “Shayan” and the diameter of the root head of SDL grew considerably, while no significant growth was observed afterwards. From the third year, the yellow–white cross-sections became more distinct. In summary, it was clear that SDL grew more rapidly in the first three years and then gradually slowed down.

### 2.2. Effect of Growth Years on Drying Rate and Methanol Extract, and Total Sterols and Total Flavonoids Contents of SDL

Based on the results of the drying rate measurement, the drying rate of SDL increased with its growth and reached a maximum of 27.05% after 3 years of cultivation, before gradually decreasing. The content of methanol extract of 1 to 5 growth years with SDL all exceeded the content specified in the ‘Pharmacopoeia of the People’s Republic of China’ (20%) and had a tendency toward that decrease with increasing growth years. The lowest content of methanol extract was 31.33% in 5-year-old SDL, significantly lower than that in 1-to-4-year-old SDL. The total flavonoids content of 3-year-old SDL was significantly higher than that of 4- and 5-year-old SDL, with the highest total flavonoids content of 2.22 g·kg^−1^, followed by that of 1- and 2-year-old SDL. In addition, the total sterols content of 3-year-old SDL was the highest (2.33 g·kg^−1^), which was significantly higher than that of 4- and 5-year-old SDL, and then 4- and 5-year-old SDL were significantly higher than that of 2- and 1-year-old SDL, and the total sterols content of 1-year-old SDL was the lowest (1.61 g·kg^−1^) (Figure 2B).

### 2.3. SDL Metabolites’ Testing Quality Control and Composition Identification

As shown in Supplementary Figure S1A,B, the peak response intensities and retention times of the chromatographic peak of the total ion chromatogram (TIC) for all QC samples largely overlap, indicating good instrument precision. The multivariate control chart (Supplementary Figure S1C,D) showed that the fluctuations of QC samples are within plus or minus three standard deviations, demonstrating that the instrument’s variations are within a normal range and that it has adequate stability. Principal component analysis (PCA) (Supplementary Figure S1E,F) showed that the QC samples were tightly clustered together, indicating good sample reproducibility. In addition, the principal component analysis also showed that the principal component scores of the samples with the same growth years were similar and closely clustered; the principal component scores of the 2-, 3-, and 4-year-old samples were similar, while the principal component scores of the 1- and 5-year-old samples were largely different from those of the other samples. A total of 1586 substances were identified by searching the local self-built standard database of Shanghai Applied Protein Technology for the identification of SDL metabolites. Among them, 880 species were identified in positive mode and 706 species in negative mode. A total of 1586 species were classified into 13 classes and more than 50 sub-classes (Table 1 and Supplementary Table S1).

### 2.4. Analysis of SDL Co-Expression Metabolites in Different Growth Years

**Figure 3:**
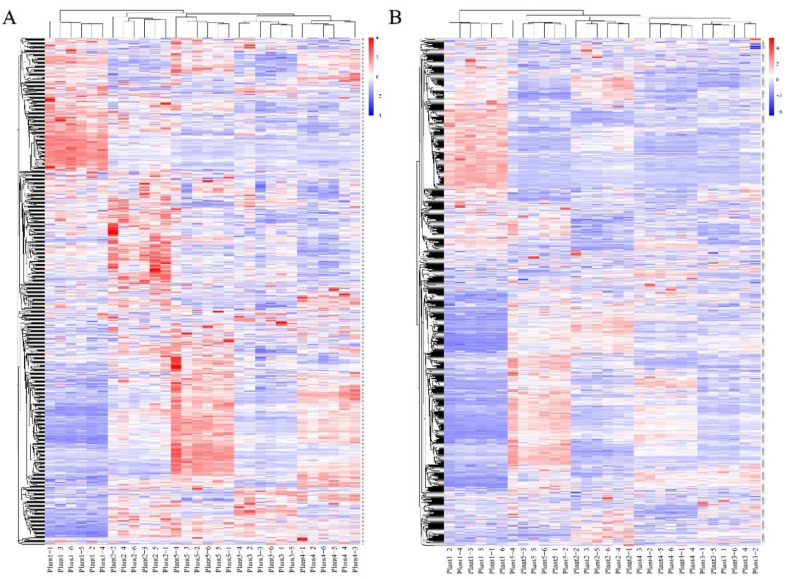
Cluster heatmap analysis of metabolites in different growth years of SDL. (**A**) Metabolites detected in positive mode; (**B**) metabolites detected in negative mode.

**Figure 4:**
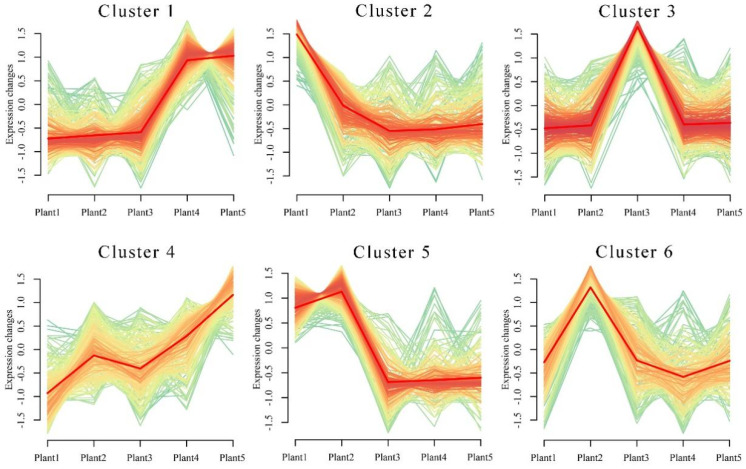
Co-expression metabolite clusters of SDL metabolites in different growth years.

**Figure 5:**
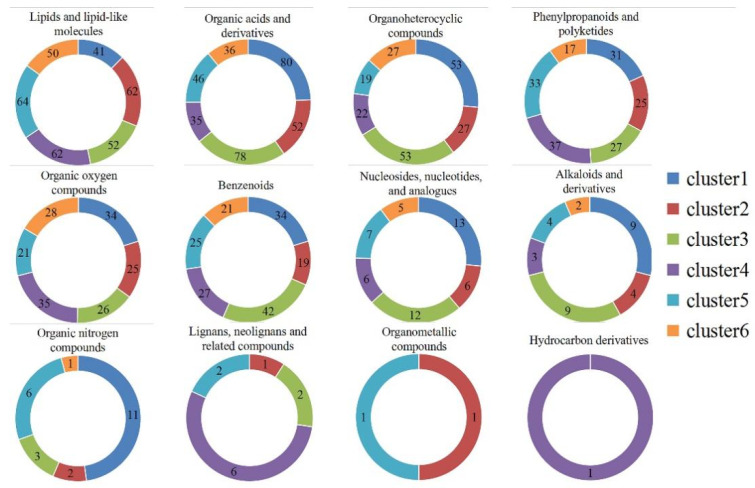
Metabolites’ classification in different metabolite clusters.

The ionic strength of all metabolites of SDL in 5 growth years was analyzed by hierarchical clustering heatmap (the raw data are shown in Supplementary Tables S2 and S3). The results show that the samples with the same growth years have similar metabolite characteristics and cluster together, while the samples with different growth years show abundant differences in metabolites, indicating that different growth years have different effects on the metabolites of SDL (Figure 3). A cluster analysis of metabolites by both positive and negative model detection grouped 2-to-5-year-old SDL into one category and 1-year-old SDL into a different category, demonstrating that the metabolites of 1-year-old SDL have greater differences. K-means-based clustering was further used to analyze the expression of all metabolites for different growth years. A total of 1586 metabolites were clustered into six co-expressed metabolite clusters (Figure 4), and each metabolite cluster represents a group of substances that show a similar trend of expression with increasing growth years. Among them, cluster 1 contained 324 metabolites, mainly reflecting substances that were co-expressed at high levels in 4- and 5-year-old SDL and showed a trend of high expression with growth years; there were 239 metabolites in cluster 2, and the high expression of 1-year-old SDL and a tendency of declining expression was demonstrated; cluster 3 contained the highest number of metabolites of 330, which showed the high expression of 3-year-old SDL; and cluster 4 comprised 245 metabolites, primarily representing the high expression of 5-year-old SDL, as well as a high expression of 2-year-old SDL. In cluster 5, there existed 246 metabolites, mainly representing substances that were commonly highly expressed in 1- and 2-year-old SDL. Cluster 6 included 202 metabolites capturing the highly expressed substances in 2-year-old SDL. The classification of metabolites in different metabolite clusters is further shown by a ring diagram (Figure 5). A high number of alkaloids and derivatives, benzenoids, nucleosides, nucleotides and analogs, organic acids and derivatives, and organoheterocyclic compounds were present in clusters 1 and 3. Organic oxygen compounds were present in greater quantities in clusters 1 and 4. There was a higher content of lipids and lipid-like molecules in clusters 2, 4, and 5. Clusters 4 and 5 contained a high number of phenylpropanoids and polyketides. Furthermore, a high number of lignans, neolignans, and related compounds were present in cluster 4.

### 2.5. Analysis of SDM for SDL of Different Growth Years

**Figure 6:**
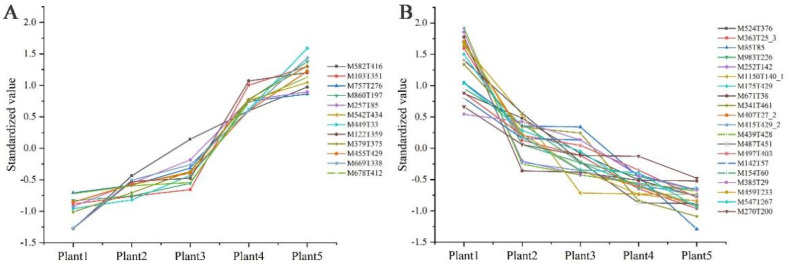
Metabolites of SDL accumulate (**A**) or decrease (**B**) with increasing growth years.

One-way ANOVA was used to analyze significantly differential metabolites (SDMs) in clusters 4 and 6, and rigorous screening was performed for metabolites that accumulated or decreased with growth years in SDL. A total of 201 and 301 metabolites in clusters 4 and 6 reached SDM levels, respectively. A total of 20 metabolites were found to decline with growth years and 12 increased, according to further screening results (Figure 6).

**Figure 7:**
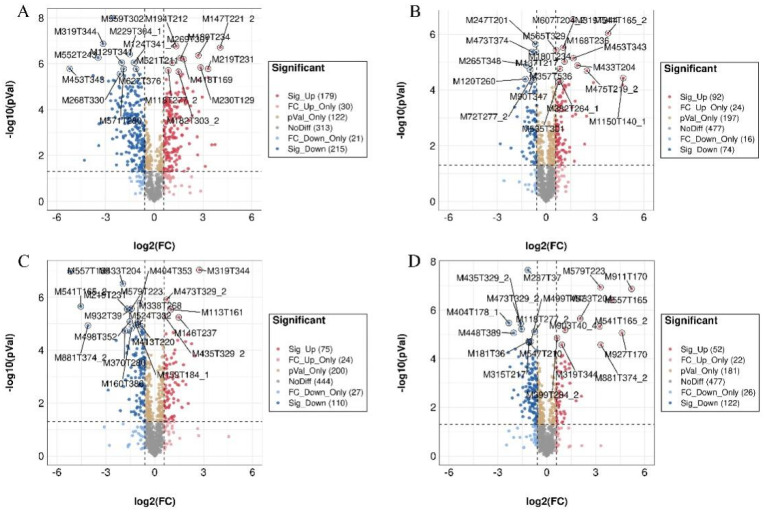
Volcano diagram of differential metabolites from different growth years of SDL (pos). (**A**) Plants 3 vs. 1; (**B**) plants 3 vs. 2; (**C**) plants 3 vs. 4; (**D**) plants 3 vs. 5.

In order to further analyze the metabolism characters and high expression of SDMs of triennial SDL, a fold-change analysis (FC analysis) and T-test were used to conduct an univariate statistical analysis of the metabolites. FC < 0.67 or FC > 1.5 and *p*-values < 0.05 were used as indicators to screen the SDMs between 3-year-old SDL and four other growth years. The results are shown in the volcano plot in Figure 7 and Supplementary Figure S2; 340 substances were significantly up-regulated in plants 3 vs. 1 (pos, 139; neg, 201) and 326 substances were significantly down-regulated (pos, 132; neg, 194); 97 substances were significantly up-regulated in plants 3 vs. 2 (pos, 22; neg, 75) and 249 substances were significantly down-regulated (pos, 151; neg, 98); plants 3 vs. 4 had 149 substances significantly up-regulated (pos, 70; neg, 79) and 249 substances significantly down-regulated (pos, 132; neg, 117); and plants 3 vs. 5 had 111 substances significantly up-regulated (pos, 42; neg, 69) and 394 substances were significantly down-regulated (pos, 189; neg, 205).

In addition, orthogonal partial least-squares discriminant analysis (OPLS-DA) was used to construct the OPLS-DA model for the multidimensional statistical analysis of metabolites. As shown in Supplementary Figures S3 and S4, the OPLS-DA model completely distinguished the two comparison samples, and the substitution test showed that all the Q2 scores were higher than 0.5, indicating that the model was stable and reliable. The variable weight value (VIP value) obtained by the OPLS-DA model was greater than 1 as an indicator to screen differential metabolites. Among them, plants 3 vs. 1 screened 397 metabolites with VIP > 1 (pos, 225; neg, 172); 353 metabolites (pos, 184; neg, 169) were screened by plants 3 vs. 2; 353 metabolites (185 in pos mode, 168 in neg mode) were screened by plants 3 vs. 4; and 369 metabolites (pos, 202; neg, 167) were screened by plants 3 vs. 5.

**Figure 8:**
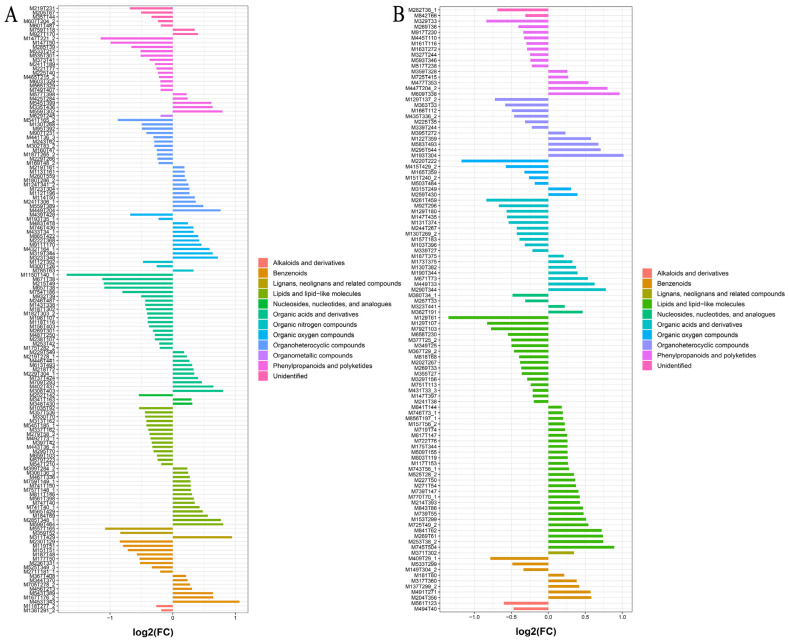
FC analysis histogram of SDMs in plants 3 vs. 1. (**A**) Metabolites detected in positive mode; (**B**) metabolites detected in negative mode.

**Figure 9:**
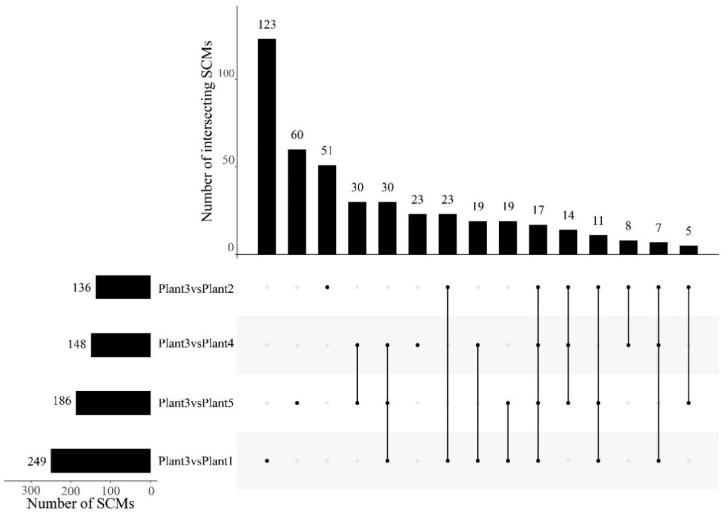
Venn diagram analysis of SDMs in different comparison groups.

**Figure 10:**
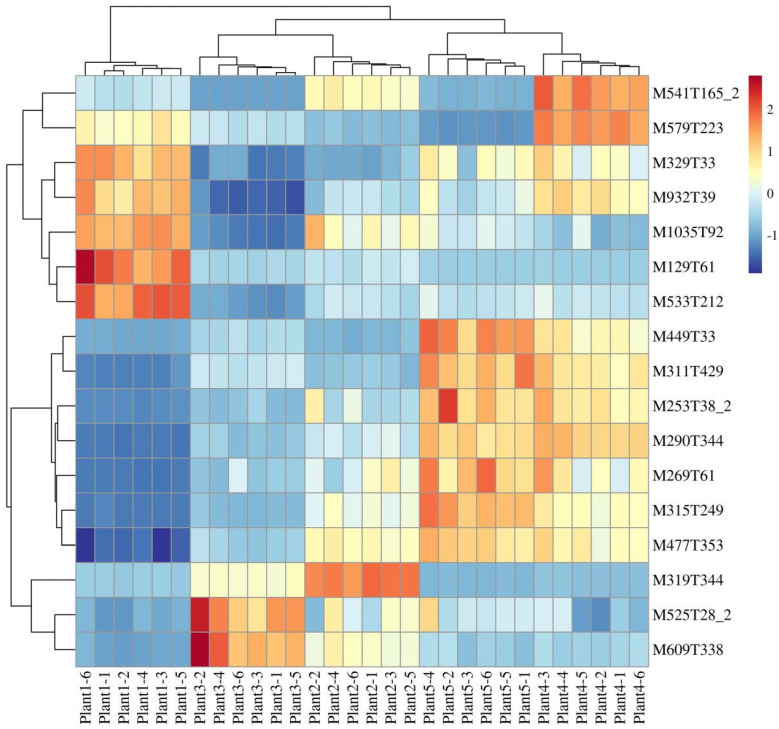
Cluster heatmap of SDMs in different growth years of SDL.

The FC analysis, *t*-test, and OPLS-DA analysis were combined to further screen for SDM. The results show that 249 SDMs are screened by plants 3 vs. 1 (pos, 142; neg, 107), 136 by plants 3 vs. 2 (pos, 62; neg, 74), 148 by plants 3 vs. 4 (pos, 70; neg, 78), and 186 by plants 3 vs. 5 (pos, 88; neg, 98). As shown in Figure 8 and Supplementary Figure S5, the FC histogram further reflects the expression and classification of SDMs of SDL at 3 years of cultivation versus other years. Based on this, a Venn diagram was used to analyze the intersection of SDMs. The results show that 17 metabolites are SDMs shared between 3-year-old SDL and other growth years (Figure 9). The hierarchical clustering analysis of the ionic strength of the 17 SDMs demonstrated that 2 substances (M525T28_2 and M609T338) were significantly higher in plant 3 than that of other growth years (Figure 10). In addition, there was a high quantity of SDMs in plants 3 vs. 1 and plants 3 vs. 5, which are 123 and 60 species, respectively, followed by plants 3 vs. 2 with 51 species and plants 3 vs. 4 with fewer unique SDMs with 23 species.

### 2.6. KEGG Pathway Enrichment Analysis of Different Metabolite Clusters

**Figure 11:**
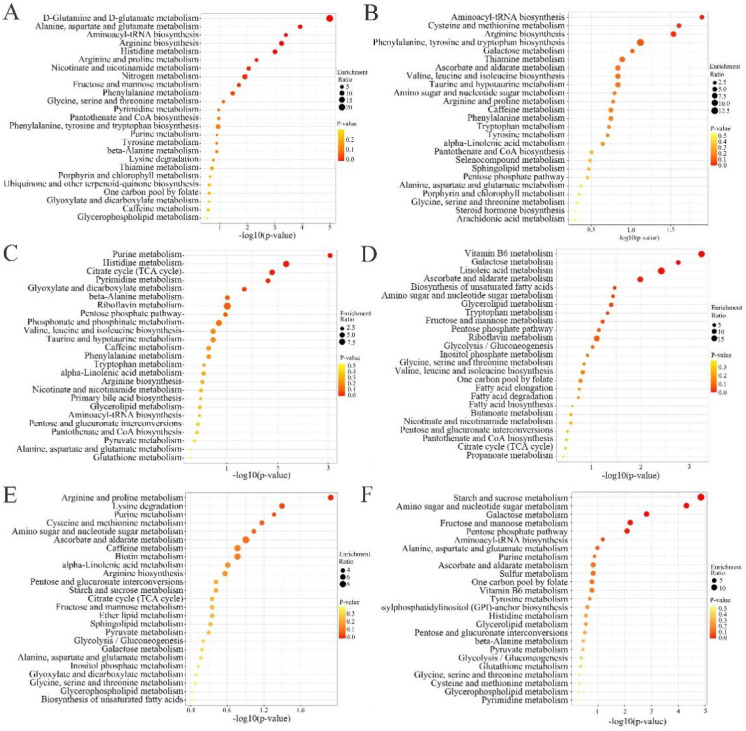
Enrichment analysis of KEGG pathway values of different metabolite clusters. (**A**) Cluster 1; (**B**) cluster 2; (**C**) cluster 3; (**D**) cluster 4; (**E**) cluster 5; (**F**) cluster 6.

The metabolites from each of the six clusters were enriched in the KEGG pathway, and the findings reveal that the metabolites from different clusters are enriched in different signaling pathways (Figure 11). Among them, cluster 1 was mainly enriched in d-glutamine and d-glutamate metabolism, alanine, aspartate and glutamate metabolism, aminoacyl-tRNA biosynthesis, arginine biosynthesis, histidine metabolism, arginine and proline metabolism, nicotinate and nicotinamide metabolism, nitrogen metabolism, fructose and mannose metabolism, and phenylalanine metabolism signaling pathways. Cluster 2 was mainly enriched in aminoacyl-tRNA biosynthesis, cysteine and methionine metabolism, and arginine biosynthesis. Cluster 3 was mainly enriched in purine metabolism, histidine metabolism, tricarboxylic acid cycle (TCA cycle), pyrimidine metabolism and glyoxylate, and dicarboxylate metabolism. Cluster 4 was mainly enriched in vitamin B6 metabolism, galactose metabolism, linoleic acid metabolism, ascorbate and aldarate metabolism, biosynthesis of unsaturated fatty acids, amino sugar and nucleotide sugar metabolism, sugar and nucleotide sugar metabolism, glycerolipid metabolism, and tryptophan metabolism. Cluster 5 was mainly enriched in arginine and proline metabolism, lysine degradation, and purine metabolism. Cluster 6 was mainly enriched in starch and sucrose metabolism, amino sugar and nucleotide sugar metabolism, galactose metabolism, fructose and mannose metabolism, and pentose phosphate pathway. This indicates that growth years not only affect the expression and accumulation of SDL metabolites, but also have an effect on the metabolic signaling pathways.

## 3. Discussion

The main traits (“Zhenzhupan”, “Sha yan”, yellow–white cross-sections) of SDL and its root head diameter were found to considerably vary sharply from 1 to 3 years after planting, while after the third year, the change slowed down. This indicates that SDL grows more rapidly from one-to-three years of planting. The drying rate can reflect the moisture and dry matter storage of medicinal materials from the side, and it is an important character determining the yield of medicinal materials. The increase in drying rate with growth years suggests that the water content proportion in SDL gradually decreased, while the dry matter content continuously accumulated, which was conducive to increasing the yield of single SDL. Methanol extract is the only content determination index specified in *Chinese Pharmacopoeia*. In this study, it was found that the content of methanol extract in SDL with five different growth years exceeded 1.5 times the specified standard, and the methanol extract content decreased continuously with the increase in growth years. This may have been caused by the increase in lignification and decrease in alcohol-soluble substances of the roots of SDL as time passed, which was similar to the patterns exhibited by *Callicarpa kwangtungensis* [15] and *Dendrobium officinale* [16]. Furthermore, the contents of total sterols and total flavonoids are the most commonly used indicators to evaluate the quality of SDL at present. Total flavonoids and total sterols content measurements of SDL in different growth years revealed that the maximum content was discovered in 3-year-old SDL, which is compatible with the findings of the research by Wang Xiufen [17] and Zhang Xueliang [18]. In addition, it was also found that rutin content in 3-year-old SDL was significantly higher than that in other growth years. The increase in growth years can therefore be shown to promote the growth of SDL features and enhance yield and drying rates; however the effect on SDL quality and component content was not totally positive.

Metabolites are the expression of the physiological state of plant life forms at the metabolic level, and their synthesis and accumulation are influenced by complex and diverse metabolic pathways and networks, and exhibit temporal and spatial dynamics [19,20]. Changes in the growth years of medicinal plants affect a series of physiological and ecological processes, such as nutrient uptake and biomass redistribution, by affecting the accumulation of metabolite substances and causing changes in plant morphology and herb yield and quality at the same time [21,22]. For example, the content of primary metabolites, such as sucrose, fructose, and choline, decreased while the content of glycine and cottonseed sugar increased as the growth period of *Polygala tenuifolia* increased; the content of secondary metabolites, such as onjisaponin Fg, polygalasaponin XXVIII, and polygalasaponin XXXII, increased, while the contents of tenuifoliose III, A, C, C2, and H were reduced [23]. The root system of three-year-old *Panax notoginseng* was the most vigorous, and the underground biomass and saponin content of three-year-old *Panax notoginseng* reached the highest level in December [21]. The accumulation of ginsenosides was not positively correlated with the growth period. It tended to increase from 3 to 5 years; however, the total ginsenoside content decreased at six years old. However, the growth period had no significant effect on the total protein content [24,25,26].

In this study, a metabolomic analysis showed that the different growth years of SDL exhibited rich metabolite diversity, and the greater the difference in growth years, the greater the metabolite differences. Plant 1 showed more significant metabolite differences with the other four growth years; plants 4 and 2 were close to plant 3 and had more similar metabolite characteristics. The number of different class metabolites in the metabolite clusters with different expression trends also differed greatly. It showed that a 1-to-2-year-old timespan was beneficial for the accumulation of more lipids and lipid-like molecules in SDL, and 3 to 5 years was beneficial for the accumulation of more alkaloids and derivatives, benzenoids, nucleosides, nucleotides and analogs, organic acids and derivatives, and organoheterocyclic compounds. Furthermore, from cluster 1, 12 SDMs were screened for an accumulation with increasing growth years, and from cluster 2, 20 SDMs were screened for a reduction with increasing growth years. Furthermore, 17 SDMs were found to be differentially expressed in 3-year-old SDL in any other year. The abovementioned results suggest that different growth years affect the accumulation of metabolites in SDL. In addition, among these SDMs, there were secondary metabolites with biological activities, such as tomatine (M1035T92), cucurbitacin e (M579T223), 4,2’-dihydroxy-3,4’,6’- trimethoxychalcone (M329T33), isorhamnetin-3-o-glucoside (M477T353), rutin (M609T338), phlorizin (M459T233), verproside (M497T403), and methotrexate (M455T429), which indicated some differences in the quality and efficacy of SDL herbs in different growth years.

The KEGG pathway enrichment analysis of co-expressed metabolite clusters showed that clusters 1 and 2 were enriched in aminoacyl-tRNA biosynthesis and arginine biosynthesis pathways. In contrast, metabolites in cluster 1 showed a gradual increase in expression levels with an increase in growth years. In addition to these two metabolic signaling pathways, some metabolites in cluster 1 were also enriched in cysteine and methionine metabolism pathways. Cysteine and methionine are important amino acids in protein composition, and methionine can be converted to cysteine. The synthesis of cysteine enhances the resistance of plants to oxygen stress [27,28]. Moreover, cysteine is central to plant sulfur metabolism, and most sulfur-containing metabolites are derived directly or indirectly from cysteine [29]. Therefore, the sulfur metabolism process may gradually increase with the increase in growth years. The expression of metabolites in cluster 2 showed a downward trend with the increase in growth years. Compared with cluster 1, cluster 2 was enriched in more abundant metabolic signaling pathways. These include nitrogen metabolism, as well as D-glutamine and D-glutamate metabolism, arginine metabolism and alanine, and aspartate and glutamate metabolism, which are amino acid metabolism pathways closely related to plant nitrogen metabolism. These metabolic pathways can generally enhance a plant’s resistance to biotic and abiotic stresses [30,31]. Additionally, these substances were enriched in fructose and mannose metabolism and nicotinate and nicotinamide metabolism. This indicated that a shorter duration of planting was conducive to the accumulation of substances, such as amino acids, sugars, and niacin.

Cluster 3 is a metabolite cluster highly expressed in 3-year-old SDL. These metabolites are not only enriched in amino acid-related metabolic pathways, but also in other metabolic pathways, such as TCA, glyoxylate, and dicarboxylate metabolism. Among them, TCA is the final metabolic pathway of sugar, lipid, and amino acid metabolism, and is a vital pathway of energy metabolism [32]. The glyoxylate and dicarboxylate metabolism pathways mainly balance local metabolic disorders in plants when they encounter external adverse disturbances and transport energy to enhance resistance [33,34]. Both metabolic pathways are closely related to plant energy metabolism, which can reflect whether the plant is growing vigorously. Therefore, the enrichment of cluster-3 metabolites into these metabolic pathways further indicates the most vigorous growth state of triennial SDL. In conclusion, the growth state and metabolic processes of SDL showed different characteristics and trends when it grew for a different number of years, which might have been the reason for the diversity of metabolites of SDL in different years.

In addition, changes in the growth years may have an impact on the accumulation of SDL metabolites due to alterations in the growth environment, particularly the soil microbial environment. When we investigated SDL planting in Tongxin County, we found that root rot was prone to occur when the growth years reached 5 years or exceeded this. Root rot disease is mostly caused by pathogenic fungi. Numerous studies have shown that growth years affect the inter-root microbial composition and diversity of medicinal herbs [28,35]. Specifically, with an increase in growth years, soil microbiota change from “bacterial” with high fertility to “fungal” with low fertility, and the trend of “fungalization” increases the number of pathogenic fungi [36]. At the same time, the abundance of some beneficial bacteria decreases and the abundance of pathogenic bacteria gradually increases, further increasing the morbidity rate [37]. For example, the number of inter-root bacteria and total number of microorganisms showed an overall trend of first increasing and then decreasing with the increase in the growth years of *Fritillaria taipaiensis*, in which the overall trend of potassium-dissolving bacteria decreased and the overall trend of fungi increased [38]. A gradual imbalance in the inter-root microbiota of *Panax ginseng* caused by an increase in growth years would cause the abundance of pathogenic fungi genus to rise and the abundance of helpful fungi genus to fall, increasing the risk of disease in *Panax ginseng* [39]. During the culture of rhizoma atractylodis, the abundance of bacterial communities decreased with growth years and the abundance of fungal communities grew; however, the number of potentially harmful bacteria increased [40]. Thus, the root rot of SDL may be caused by the degradation of the soil’s microbiome as a result of increased planting.

Meanwhile, changes in the soil microbial environment can have a significant effect on the growth and development of medicinal plants and the accumulation of secondary metabolites [41,42]. It has been shown that *Bacillus*, *Lysinibacillus*, *Rhizobium*, *Stenotrophomonas*, *Erwinia*, *Ochrobactrum*, *Enterobacter*, and *Pantoea* largely increase the root vigor and ginsenoside content of *Panax ginseng* [43]. *Artemisia annua’s* volatile oil and artemisinin content can both be considerably increased by inoculation with mycorrhizal fungi from the cluster [44]. The increase in the number of *Moses’ Ballooning mold* can promote the synthesis and accumulation of phellodendrin, berberine, and pharmacophorine in *Phellodendron Bark* [45], and also facilitate the synthesis and accumulation of flavonoids in *Astragalus* [46]. Therefore, in addition to their own development and substance accumulation patterns, the notable variations in SDL metabolites in different cultivation years may be strongly related to the changes in the microbial environment, which merits our attention and further study.

In summary, combined with the existing evaluation indicators, it can be determined that 3 plant years is the best harvest period for SDL. Compared with other growth years, 3-year-old SDL has the highest total sterols and total flavonoids contents while achieving a higher yield. In addition, the effects of growth years on the metabolites of SDL were significant and complex. This study found that SDL with different growth years contained many functional SDMs. These SDMs may further affect the efficacy of SDL, which needs further study, and it can be used as substances to focus on the exploration of the quality markers of SDL in the future.

## 4. Materials and Methods

### 4.1. Sample Collection

SDL roots were collected from 1-to-5-years-old plants named plants 1~5, respectively. All samples were collected in September 2020 at the SDL production plantation in Tongxin County, Wuzhong City, Ningxia Hui Autonomous Region (latitude 36.76° N, longitude 106.36° E, altitude 1558.00 m), and were identified as *Stellaria dichotoma* L. var. *lanceolata* Bge. The number of biological replicates for each growth-year SDL sample was six. The collected samples were naturally dried to a constant weight and stored in a light-resistant and low-temperature environment. In addition, the quality control (QC) samples were prepared by mixing multiple sources of SDL to assess the precision, stability, and repeatability of the mass spectrometric detection.

### 4.2. Determination of the Characteristics, Methanol Extract, and Total Flavonoids and Total Sterols Contents

With reference to the Pharmacopoeia of the People’s Republic of China (Volume I) (2020 Edition) [2] under the ‘*Stellariae Radix*’ provisions and the characteristics of medicinal materials, samples of ‘Sand eye’, ‘Zhenzhupan’, and yellow and white cross-section characteristics were observed and compared. Detection of drying rate: the root fresh weight and weight after complete drying were measured by the weighing method, and then the drying rate was calculated according to the following formula: drying rate = dry weight/fresh weight × 100%. The content determination of methanol extract, and total flavonoids and total sterols contents was based on the previously reported methods [47].

### 4.3. Metabolomics Analysis

Metabolomics analysis adopted the previous method we reported [47]. The medicinal powder was ground in liquid nitrogen, and 200 mg was weighed in a 2 mL centrifuge tube, added to 70% methanol solution for extraction, then vacuum dried and stored at −80 °C for later use. Before the use of the machine, 40% acetonitrile solution was added for reconstitution, fully vortexed, centrifuged at 14,000 r for 10 min, and the supernatant was obtained for metabolic component analysis. The separation was performed on an Agilent 1290 Infinity LC HILIC column. Mass spectrometry analysis was performed using a triple TOF 6600 mass spectrometer with an electrospray ionization source (ESI) with positive and negative ion (pos and neg)-mode detection. The metabolites were identified by searching the local self-built standards database established by Shanghai Applied Protein Technology Co., Ltd., Shanghai, China, and the information of retention time, molecular weight (error < 25 ppm), secondary fragmentation spectrum, and collision spectrum of metabolites were matched [47].

### 4.4. Data Statistics and Analysis

Principal component analysis (PCA), fold-change analysis (FC), *t*-test, orthogonal partial least-squares discriminant analysis (OPLS-DA), cluster heatmap analysis, volcano chart analysis, Wayne chart analysis, metabolite co-expression trend analysis, and KEGG pathway enrichment analysis were performed on the relevant data by using statistical analysis software, such as Excel 2020, SPSS Statistics 22, Origin 2018, R software 3.6.1, and online analysis platforms, such as Zhongke New Life Bioinformatics Cloud Platform and Metabo Analyst 5.0.

## 5. Conclusions

The characteristics of the medicinal materials and drying rate of SDL showed a positive trend with increasing growth years, with the fastest development period occurring 3 years after planting, followed by a gradual decrease. The third year of cultivation was found to be the most favorable harvesting year for SDL, exhibiting high refractive dryness, methanolic leachate content, and the maximum total sterols and total flavonoids contents. Metabolomics identified a total of 1586 metabolites, classified into 13 classes with more than 50 sub-classes. The results of the multivariate statistical analysis demonstrate that growth years significantly contribute to the formation and accumulation of SDL metabolites, with marked differences being observed for varying growth years. Additionally, highly expressed metabolites in SDL differed by growth years, whereby the accumulation of more lipids occurred during the first two years and more alkaloids and benzenoids during years 3–5. After screening, 12 metabolites were identified as accumulating with growth years, 20 as decreasing with growth years, and 17 as significantly differentially expressed in 3-year-old SDL, thereby indicating their potential as bioactive substance quality markers for SDL. In summary, this study revealed that SDL metabolites are significantly impacted by growth years, reflecting the growth and accumulation patterns of metabolites and their functional components. Furthermore, the study identified the most favorable harvesting year for SDL, providing a reference for high-yield and high-quality production.

## Figures and Tables

**Table 1:** Classification table for SDL metabolites. The number in “( )” represents the number of metabolites.

No.	Class	Sub-Class
1	Lipids and lipid-like molecules (331)	Fatty acyls (112), glycerophospholipids (81), prenol lipids (78), steroids and steroid derivatives (48), sphingolipids (7), glycerolipids (5)
2	Organic acids and derivatives (327)	Carboxylic acids and derivatives (282), hydroxy acids and derivatives (15), peptidomimetics (8), keto acids and derivatives (7), organic phosphoric acids and derivatives (5), organic sulfonic acids and derivatives (5), others (5)
3	Organoheterocyclic compounds (201)	Indoles and derivatives (43), pyridines and derivatives (19), benzopyrans (15), imidazopyrimidines (14), azoles (13), quinolines and derivatives (12), diazines (11), others (74)
4	Phenylpropanoids and polyketides (170)	Flavonoids (86), cinnamic acids and derivatives (24), isoflavonoids (15), coumarins and derivatives (17), diarylheptanoids (8), linear 1,3-diarylpropanoids (7), stilbenes (5), tannins (5), phenylpropanoic acids (3)
5	Benzenoids (168)	Benzene and substituted derivatives (108), phenols (44), naphthalenes (9), anthracenes (4), indenes and isoindenes(2), pyrenes (1)
6	Nucleosides, nucleotides, and analogues (49)	Purine nucleosides (9), pyrimidine nucleotides (10), purine nucleotides (11), pyrimidine nucleosides (7), others (12)
7	Alkaloids and derivatives (31)	Pyrrole alkaloid (13), isoquinoline alkaloid (8), indole alkaloids (2), quaternary ammonium hydroxide (3), ergot alkaloid (2), others (3)
8	Lignans, neolignans, and related compounds (11)	Furanoid lignans (6), dibenzylbutane lignans (2), lignan lactones (3)
9	Organometallic compounds (2)	Organometalloid compounds (1), organo-post-transition metal compounds (1)
10	Organic oxygen compounds (169)	Organic oxygen compounds (169)
11	Organic nitrogen compounds (23)	Organic nitrogen compounds (23)
12	Hydrocarbon derivatives (1)	Hydrocarbon derivatives (1)
13	Unidentified (103)	Unidentified (103)
14	Total (1586)	Total (1586)

## Data Availability

Data is contained within the article or Supplementary Materials.

## Supplementary Materials

The following supporting information can be downloaded at: https://www.mdpi.com/article/10.3390/plants12122286/s1. Additional data relevant to this paper can be found in the Annexes. Supplementary Figure S1. Quality control analysis of SDL metabolites detection. A and B: total ion chromatograms of QC samples, shown as overlap diagrams in pos and neg modes. C and D: multivariate control chart in pos and neg modes. E and F: PCA of all samples in pos and neg modes. Supplementary Figure S2. Volcano diagram of differential metabolites from different growth years of SDL (neg). A, Plants 3 vs. 1; B, plants 3 vs. 2; C, plants 3 vs. 4; D, plants 3 vs. 5. Supplementary Figure S3. OPLS-DA and permutation test analysis of differential metabolites of different comparison groups (pos). A, C, E, and G: OPLS-DA and permutation test analysis for detecting metabolites; B, D, F, and H: permutation test analysis for detecting metabolites. Supplementary Figure S4. OPLS-DA and permutation test analysis of differential metabolites of different comparison groups (neg). A, C, E, and G: OPLS-DA and permutation test analysis for detecting metabolites; B, D, F, and H: permutation test analysis for detecting metabolites. Supplementary Figure S5. FC analysis histogram of SDMs in different growth years. A and B: plants 3 vs. 2 in pos and neg modes, C and D: plants 3 vs. 4 in pos and neg modes, E and F: plants 3 vs. 5 in pos and neg modes. Supplementary Table S1. Basic information of metabolites in SDL. Supplementary Table S2. Raw data of SDL metabolites in different growth years (pos). Supplementary Table S3. Raw data of SDL metabolites in different growth years (neg).
