# Supplementary material for: The Impact of Growth Years on the Medicinal Material Characteristics and Metabolites of Stellaria dichotoma L. var. lanceolata Bge. Reveals the Optimal Harvest Age
Source: Plants (Basel). 2023 Jun 12;12(12):2286. doi: 10.3390/plants12122286 (PMC10304078; doi:10.3390/plants12122286)
Supplement: Supplementary file 1 [file plants-12-02286-s001.zip › Supplementary Table S1 Basic information of metabolites in SDL.pdf]

Supplementary Table S1 Basic information of metabolites in YCH

ID	Name	ID	Name
M494T40	alpha.-ergocryptine	M147T54	Myosmine
M481T266	2,3-dehydrosilybin a	M149T137	N-benzylacetamidine
M561T123	Protoporphyrin ix	M181T349_1	3,4-dihydroxyhydrocinnamic acid
M592T217	Ergoloidmesylat	M217T180	Tetrahydroharmine
M181T170	Harmane	M219T210	8-acetyl-7-methoxycoumarin
M609T263_1	Reserpine	M316T415	Lycopsamine n-oxide
M149T163	Stylopine	M366T194	Isopentenyl-adenine-7-glucoside
M149T262	trans-cinnamate	M401T243	Aloin
M274T213	Emetine	M414T457_2	Echimidine n-oxide
M301T154	Etifoxine	M514T332	Harringtonine
M312T57	Norisoboldine	M118T277_2	Betaine
M325T28	Hydroquinidine	M208T197	Phenylalanine betaine
M575T229	2-[[7-hydroxy-1-(4-hydroxy-3,5-dimethoxyphenyl)-3-(hydroxymethyl)-6,8-dimethoxy-1,2,3,4-tetrahydronaphthalen-2-yl]methoxy]oxane-3,4,5-triol	M276T483	.gamma.-L-Glu-.epsilon.-L-Lys
		M498T352	Codeine-6-.beta.-d-glucuronide
		M247T201	Tryptophan betaine
M138T291_2	Trigonelline	M376T34_2	Tuberostemonine
M142T117	N-phenyl-1-naphthylamine	M245T187_1	Ile-Met
M271T181_1	Aloe emodin	M423T205	Candesartan cilexetil
M367T218_2	Hirsuteine	M429T340	Ketoprofen .beta.-d-glucuronide
M491T167	Aurantio-obtusin beta-d-glucoside	M431T109_1	Trifloxystrobin
M499T500	13,14-epoxyfluprostenol isopropyl ester	M438T30	5-hexenoic acid, 6-[(2r,3s)-3-[[[(4-chloro-2-methylphenyl)sulfonyl]amino]methyl]bicyclo[2.2.2]oct-2-yl]-, (5z)-
M103T260	Phenylacetaldehyde		
M439T375	Candesartan		
M105T176	Phenylethylamine	M453T343	2-pyrimidinecarboxamide, hexahydro-n-hydroxy-1,3-bis[(4-methoxyphenyl)sulfonyl]-5,5-dimethyl-
M110T52	4-aminophenol		
M466T333	Propaquizafop		
M105T38	4-methylbenzyl alcohol	M456T213	Octanoic acid, 2-[[4-([1,1'-biphenyl]-4-ylamino)-6-chloro-2-pyrimidinyl]thio]-
M123T242	Benzoic acid		
M117T201	Chlordimeform	M491T271	Propanoic acid, 2-[[4-[2-[[[(2,4-difluorophenyl)amino]carbonyl]heptylamino]ethyl]phenyl]thio]-2-methyl-
M119T221	4-methylhippuric acid		
M497T282_1	Dapsone		
M119T51	P-toluamide	M504T340	Ampkinone
M120T260	Tyramine	M507T285	2,4,11,13-tetraazatetradecanediamide, n1,n14-bis(4-chlorophenyl)-3,12-diimino-
M513T179	Glimepiride		
M125T176	Hydroxyhydroquinone	M515T407	Telmisartan
M125T230	Fenpropathrin	M524T376	Penfluridol
M126T105	3-hydroxyacetaminophen	M525T349_3	Benzenepropanoic acid, 5-(3-carboxybenzoyl)-2-[[(5e)-6-(4-methoxyphenyl)-5-hexen-1-yl]oxy]-
M133T34	2-methylamino-1-phenylbutane		
M529T205	Trans-hydroxyglimepiride		
M134T135	2-amino-4-methylbenzophenone	M543T389	Trans-carboxyglimepiride

M136T30	Anthranilic acid (Vitamin L1)	M547T278_2	Antimycin a
M136T98	3-hydroxyanthranilic acid	M549T375_2	Benzoylnorecgonine
M136T99_2	4-aminobenzoate	M582T416	Benzeneacetic acid, 3-[3-[[[2-chloro-3-(trifluoromethyl)phenyl]methyl](2,2-diphenylethyl)amino]propoxy]-
M137T299_2	4-hydroxybenzoate		
M583T232	Crufomate	M648T410	Pyribenzoxim
M137T34	Salicylic acid		
M149T304_2	3-methylphenylacetic acid	M655T412	2-thiophenecarboxamide, n-[[4'-[[3-butyl-1,5-dihydro-5-oxo-1-[2-(trifluoromethyl)phenyl]-4h-1,2,4-triazol-4-yl]methyl][1,1'-biphenyl]-2-yl]sulfonyl]-3-methyl-
M149T31	Monobutyl phthalate		
M671T497	Fenamiphos sulfone	M749T238_1	Erioglaucine
M153T101	Gentisic acid		
M153T289	3,4-dihydroxybenzoic acid	M793T191	Phosphonic acid, p-[[4-[(1-oxotetradecyl)amino]phenyl]methyl]-
M155T300	Niclosamide	M94T262	Aniline
M164T64_2	Formylanthranilic acid	M681T285	Indanofan
M167T135	2,3-dihydroxybiphenyl	M713T346	Sulindac
M167T176_2	Dioxacarb	M155T140	1-naphthoic acid
M167T333_2	Vanillic acid	M160T135_2	2-amino-1-naphthol
M168T289	2-methoxy-5-nitrophenol	M161T137_1	5,8-dihydroxy-1,4-naphthoquinone
M177T50	Icilin	M171T66	1-naphthalenecarboxaldehyde, 2-hydroxy
M180T173	P-acetaminobenzoic acid	M175T241	Juglone
M181T163_2	Karbutilate	M215T65	2,3-naphthalenedicarboxylic acid
M181T54	Methyl orsellinate	M367T408	Atovaquone
M182T366	Pargyline	M555T420	Didansyl-1,4-diaminobutane
M183T66	5-hydroxyisovanillic acid	M573T212_3	Oleana-1,9(11)-dien-28-amide, 2-cyano-3,12-dioxo-n-(2,2,2-trifluoroethyl)-
M208T160	Benzoylcholine	M108T52	3-nitrophenol
M187T48	Tetrahydromagnolol		
M189T107	2,4-dichlorobenzoic acid	M136T36	2-hydroxy-3-methoxybenzaldehyde
M197T44	Homoveratric acid	M137T172_2	4-hydroxy-3-methoxybenzyl alcohol
M199T51_1	4-nitrosodiphenylamine	M149T440	Dl-4-hydroxy-3-methoxymandelic acid
M204T356	1h-imidazol-1-yloxy, 2-(4-carboxyphenyl)-4,5-dihydro-4,4,5,5-tetramethyl-, 3-oxide	M151T119	Synephrine
M213T20	2,4-dihydroxybenzophenone	M151T36	Vanillin
		M151T51	3-methoxytyramine
M213T45	2,4,5-trimethoxybenzoic acid	M154T429	Fludarabine
M217T151	Chlorophene	M165T141	3,4-dihydroxymandelic acid
M219T182	Hydroxyebastine	M165T37	3-Methoxy-4-hydroxyphenylethyleneglycol
M221T75	Monoisobutyl phthalate	M165T73	Vanylglycol
M225T139	Isopropyl 4-hydroxybenzoate	M168T479	6-Hydroxydopamine

M230T129	3,3'-dimethoxybenzidine	M171T168	Resorcinol
M236T331	1-(benzo[d][1,3]dioxol-4-yl)-2-(methylamino)pentan-1-one	M177T36	Coniferyl aldehyde
M255T175_2	Ketoprofen	M179T151	Coniferyl alcohol
M265T26	Zinniol	M179T172_2	Dihydroisoferulic acid
M267T332	Valsartan acid	M179T53_1	Coniferol
M274T63	Fenpropidin	M181T259	Dihydroconiferyl alcohol
M279T32	Dibutyl phthalate	M181T80	Dl-4-hydroxyphenyllactic acid
M286T294	Letrozole	M183T27	2,4-dinitrophenol
M292T304	Cyproconazole	M194T588	Isoproterenol
M304T125	Benzalkonium chloride (c12)	M197T177	DL-Vanillylmandelic acid
M305T30	Heptanedioic acid, 1-[2-[(2-carboxyphenyl)methylene]hydrazide]	M197T26	4,6-dinitro-o-cresol
M312T168	Norcocaine	M209T266	Sinapyl alcohol
M318T304	Triticonazole	M220T84	2,6-di-tert-butyl-4-methoxyphenol
M323T204	Acetylfentanyl	M257T114	His-Cys
M324T497_1	Thenylchlor	M308T107	Dihydrocapsaicin
M337T231	Benzamide, 2-[(e)-(1,4-dimethyl-2-piperazinylidene)amino]-5-nitro-n-phenyl-	M371T35	Sulindac sulfone
M337T256	Propanoic acid, 3-[[[4-[4-[[[1-(2-chlorophenyl)ethoxy]carbonyl]amino]-3- methyl-5-isoxazolyl]phenyl]methyl]thio]-	M335T216	Benzyl butyl phthalate
M341T28	Phenol, 5-(1,1-dimethylheptyl)-2-[(1r,2r,5r)-5-hydroxy-2-(3-hydroxypropyl)cyclohexyl]-	M385T326	(-)-Medicarpin
		M365T153	Homovanillic acid
		M331T56	Butamifos
M344T370	Propiconazole	M405T143	Tyr-Phe
		M321T50	8-gingerol
		M328T419_1	Diclobutrazol
M345T154	8-(3-((2-carboxypropan-2-yl)oxy)phenyl)octanoic acid	M463T287	1.alpha.-methyl-5.alpha.-androstan-3.alpha.,17.beta.-diol glucuronide
		M493T24	Novaluron
M345T57	Oryzalin	M529T32_1	Kukoamine a
M350T394	L-tosylphenylalanyl chloromethyl ketone	M533T299	Aprepitant .beta.-glucuronide (position of glucuronide on triazolone ring uncertain)
M317T360	1,3,6-tri-o-galloyl-.beta.-d-glucose	M539T342	Selaginpulvilin o
M361T303_2	Dimefuron		
M368T387	Benzoic acid, 4-[(1s)-1-[[5-chloro-2-(4-fluorophenoxy)benzoyl]amino]ethyl]-	M540T162_2	(2r,3r,4s,5s,6r)-2-[[7-hydroxy-1-(4-hydroxy-3-methoxyphenyl)-3-(hydroxymethyl)-6-methoxy-1,2,3,4-tetrahydronaphthalen-2-yl]methoxy]-6-(hydroxymethyl)oxane-3,4,5-triol
M391T32_1	Dioctyl phthalate	M671T193_3	1-Hexadecanoyl-2-(9Z,12Z-octadecadienoyl)-sn-glycero-3-phosphoric acid
M695T31_5	S4:18(p3:16/f1:2)		
M402T290	Butachlor esa	M705T278_2	2-[4-[(3s,3ar,6s,6ar)-6-[3-methoxy-4-[3,4,5-trihydroxy-6-(hydroxymethyl)oxan-2-yl]oxyphenyl]-1,3,3a,4,6,6a-hexahydrofuro[3,4-c]furan-3-yl]-2-me
M543T356	4-methoxy-6-[2-(4-methoxyphenyl)ethyl]pyr		

	an-2-one		thoxyphenoxy]-6-(hydroxymethyl)oxane-3,4,5-triol
M404T178_1	N4-phthalylsulfathiazole	M503T403	Hypericin
M409T29_1	Bensulfuron-methyl		
M93T299	Phenol		
M421T263_1	(5-benzoyloxy-4,6-dihydroxy-3-methoxycyclohexen-1-yl)methyl benzoate	M403T183_1	Pyrazoxyfen
M440T158_1	N-trifluoroacetyl deacetylcolchiceine		
M361T180	(-)-secoisolariciresinol	M542T175	Doxorubicin
		M541T466	Cadusafos
M605T412	Enterodiol	M557T165	Arctiin
M311T429	(-)-hydroxymatairesinol	M191T68	Sedoheptulosan
M359T160	Lariciresinol	M399T388_2	4'-demethylpodophyllotoxin
M359T52	Matairesinol	M459T308	Podophyllotoxin
M371T302	Arctigenin	M253T296	2-keto-D-Gluconic acid
M330T70	Eicosapentaenoic Acid ethyl ester	M789T149	Pc 32:2
M101T212	3-hydroxyisovaleric acid	M791T185	Pe(18:1e/20-hdohe)
M103T351	Pentanoic acid	M792T103	1-hexadecyl lysophosphatidic acid
M113T392_1	(2e,4e)-hexa-2,4-dienoic acid	M796T202_1	Pg 38:5
M115T80_2	Isocaproic acid	M803T119	Pc 33:2
M117T153	2-methyl-3-hydroxybutyric acid	M807T115	1-palmitoyl-2-docosahexaenoyl-sn-glycero-3-phosphocholine
M117T262	2-Hydroxy-3-methylbutyric acid	M809T185	1,2-dioleoyl-sn-glycero-3-phosphatidylcholine
M129T107	Citraconic acid	M810T201_2	Pi 32:0
M129T61	Mesaconic acid	M811T146	1-Stearoyl-2-oleoyl-sn-glycerol 3-phosphocholine (SOPC)
M131T193	Mevalonic acid	M815T146	Pc 34:3
M141T349	Cis,cis-muconic acid	M817T147	Pc(16:1e/9-hode)
M143T60_2	Caprylic acid	M818T68	Pg 40:8
M147T397	Itaconic acid	M834T199	Pi 34:2
M157T56_2	Pelargonic acid	M835T30_1	1,2-distearoyl-sn-glycero-3-phosphocholine
M161T391_2	3-hydroxy-3-methylglutaric acid	M839T144	Pc(16:1e/8,9-epete)
M170T342	7-keto-8-aminopelargonic acid	M840T66_1	Pg 42:11
M173T361	Suberic acid	M841T144	Pc(16:0e/8-hepe)
M175T106	3-Isopropylmalate	M841T62	Pc(16:0e/11,12-epete)
M175T344	2-Isopropylmalic acid	M843T86	Pc(16:0e/5,6-eet)
M187T347_2	Azelaic acid	M848T204	Pi(16:1/13-hode)
M202T267	Acetylcarnitine	M850T203	1,2-dipalmitoyl-sn-glycero-3-phospho-(1'-myo-inositol)
M204T71	Pantothenol	M850T205	Pi(16:0/9-hode)
M227T50	Myristic acid	M856T197_1	Pi 36:5
M241T226	(z)-2-octylpent-2-enedioic acid	M857T153	Pc(16:0/8-hete)
M241T38	Pentadecanoic acid	M858T197	Pi 36:4
M245T36_2	1-monolinoleoyl-rac-glycerol	M860T197	Pi(16:0e/15-hete)

M246T243	2-methylbutyryl-l-carnitine	M875T178	1,2-diarachidonoyl-sn-glycero-3-phosphocholine
M253T38_2	Cis-9-palmitoleic acid	M884T233	Pi 38:5
M255T38	Palmitic acid	M886T161	Pi 38:4
M256T36_3	Palmitamide	M891T181	Pc 40:7
M261T187	9-(2,3-dihydroxypropoxy)-9-oxononanoic acid	M900T230	Pi(20:3/13-hode)
M263T78	Linoleic acid methyl ester	M913T207	Pc 42:10
M269T33	1-octadecanol (drug)	M215T21	Phenyl salicylate
M271T54	16-hydroxyhexadecanoic acid	M1028T248	Soyasapogenol b + dhex + hex + hexa + malonyl
M279T38	Linoleic acid	M233T30	Valerenic acid
M279T58_2	Linolenic acid	M255T58	(6e)-2,6,10-trimethyldodeca-6,11-diene-2,3,10-triol
M281T60_2	20-hydroxy-4z,7z,10z,13z,16z,18e-docosahexaenoic acid	M258T112	N-mesitylbicyclo[2.2.1]heptane-2-carboxamide
M282T35_2	Oleamide	M269T61	All-trans-4-ketoretinoic acid
M283T58	Avocadyne	M287T35	Benzene, 1,3-dimethoxy-5-methyl-2-[(1r,6r)-3-methyl-6-(1-methylethenyl)-2-cyclohexen-1-yl]-
M283T37	Octadecanoic acid	M291T264	Acetoxyvalerenic acid
M285T60	Agnuside	M297T28	Ostruthin
M285T206_2	Hexadecanedioic acid		
M285T348_1	Malathion	M297T39	Pristanic acid
M287T120_2	5z,8z,14z-eicosatrienoic acid		
M293T37	13s-hydroperoxy-9z,11e-octadecadienoic acid	M311T28	Thymol-beta-d-glucoside
M293T74	9s-hydroperoxy-10e,12z-octadecadienoic acid	M311T37	Phytanic acid
M293T79	9-hydroperoxy-10e,12z,15z-octadecatrienoic acid	M335T373	Nivalenol
M295T37	9-hydroxy-10e,12z-octadecadienoic acid	M351T181	Anisatin
M295T70	9-oxo-10e,12z-octadecadienoic acid	M353T183	Geniposide
M296T36	alpha-Linolenic acid	M360T219_2	Napelline
M297T131	Ricinoleic acid	M369T471	Aucubin
M299T103	9,10-dihydro-15-deoxy-.delta.12,14-prostaglandin j2	M373T138	Geniposidic acid
M301T35	Prallethrin	M377T25_2	5-[1,2,4a-trimethyl-5-(propanoyloxymethyl)-2,3,4,7,8,8a-hexahydronaphthalen-1-yl]-3-methylpentanoic acid
M303T31	Aleuritic acid	M405T176	8-o-acetylharpagide
M305T39	11,14,17-eicosatrienoic acid, (z,z,z)-	M409T157	.beta.-tocotrienol
M307T104	Fa 18:4+2o	M411T106_2	Ursolic acid methyl ester
M307T38	8z,14z-eicosadienoic acid	M413T220	Loganin
M309T38	Eicosenoic acid		
M311T67	Fa 18:2+2o	M421T369	Methyl (1s,4as,6r,7s,7as)-5,6-dihydroxy-7-(hydro

			xymethyl)-1-[(2s,3r,4s,5s,6r)-3,4,5-trihydroxy-6-(hydroxymethyl)oxan-2-yl]oxy-1,4a,5,6,7,7a-hexahydrocyclopenta[c]pyran-4-carboxylate
M399T284_2	Loganic acid	M425T27_3	(2e,6e,10e)-13-[(2r)-6-hydroxy-2,8-dimethyl-3,4-dihydrochromen-2-yl]-2,6,10-trimethyltrideca-2,6,10-trienoic acid
M303T39	Arachidonic acid (peroxide free)	M425T36	Lanosterol
M403T54	Oleoside 11-methyl ester	M429T30	alpha-Tocopherol (Vitamin E)
M313T172	Octadecanedioic acid		
M402T154	Sorbitane monopalmitate - polysorbate 40 in-source fragment	M431T220	Eurycomanone
M313T91	9,10-dihydroxy-12z-octadecenoic acid		
M321T52	15s-hydroperoxy-11z,13e-eicosadienoic acid		
M323T245	Maltose	M434T51_3	N-acetyl-s-geranylgeranyl-l-cysteine
M325T316	Lactitol		
M327T36	1,2-dihydroxyheptadec-16-yn-4-yl acetate	M437T34_2	Alisol b acetate
M327T52_2	12-hete-[d8]	M439T38	Roburic acid
M329T156	(z)-9,12,13-trihydroxyoctadec-15-enoic acid	M441T301	Ginkgolide c
M335T25_2	Prostaglandin b1	M445T284_1	Lamiide
M337T162	2-linoleoylglycerol	M455T106	Oleanolic acid
M337T38	Erucic acid	M463T26	Pristimerin
M339T38	Behenic acid	M467T117	Glabrolide
M341T145	Hexadecanedioic acid, 3,3,14,14-tetramethyl-	M469T307	Limonin
M344T409	Neohesperidose	M471T239	18.beta.-glycyrrhetinic acid
M347T27_2	5s,12r-dihydroxy-6z,8e,10e,14z-eicosatetraene-1,20-dioic acid	M471T297	Deoxynivalenol 3-glucuronide
M351T269	Lipoxin a4	M471T32_3	Echinocystic acid
M353T157	Prostaglandin i2	M471T355	Evodin
M353T37	Tricosanoic acid	M481T426	Albiflorin
M353T457	6:3+6o fatty acyl hexoside	M487T215	Enhydrin
M355T27	16-phenyltetranorprostaglandin f2.alpha.	M495T134	(1s,4as,7s,7as)-7-hydroxy-7-methyl-1-[(2s,3r,4s,5s,6r)-3,4,5-trihydroxy-6-[(4-hydroxybenzoyl)oxymethyl]oxan-2-yl]oxy-4a,5,6,7a-tetrahydro-1h-cyclopenta[c]pyran-4-carboxylic acid
M360T366_2	D-turanose	M497T28_3	Ganoderic acid a
M497T135	Poricoic acid a	M503T28_4	Madecassic acid
M363T25_3	9s,11r,15s-trihydroxy-20a,20b-dihomo-5z,13e-prostadienoic acid	M515T186_1	Nomilin
M364T463	Guanosine 5'-monophosphate (GMP)		
M365T38	Nervonic acid	M523T28	Asiatic acid
M371T400	Rutinose		
M377T27_2	16,16-dimethyl-6-ketoprostaglandin e1	M529T300	Harpagoside
M381T172	1a,1b-dihomoprostaglandin e1	M531T182	Phytolaccagenin

M381T400	5-heptenoic acid, 7-[(1r,2r,3s,5s)-2-[(1e,3s)-3-(2,3-dihydro-1h-inden-2-yl)-3-hydroxy-1-propen-1-yl]-3-fluoro-5-hydroxycyclopentyl]-, (5z)-	M547T210	[4a,7-dihydroxy-7-methyl-1-[3,4,5-trihydroxy-6-(hydroxymethyl)oxan-2-yl]oxy-1,5,6,7a-tetrahydrocyclopenta[c]pyran-5-yl] (e)-3-(4-methoxyphenyl)prop-2-enoate
M589T290	(4s,5z,6s)-5-[2-[(e)-3-(4-hydroxyphenyl)prop-2-enoyl]oxyethylidene]-4-(2-methoxy-2-oxoethyl)-6-[(2s,3r,4s,5s,6r)-3,4,5-trihydroxy-6-(hydroxymethyl)oxan-2-yl]oxy-4h-pyran-3-carboxylic acid	M561T398	(1s,4as,7s,7as)-1-[(2s,3r,4s,5s,6r)-6-[[(e)-3-(3,4-dihydroxyphenyl)prop-2-enoyl]oxymethyl]-3,4,5-trihydroxyoxan-2-yl]oxy-7-hydroxy-7-methyl-4a,5,6,7a-tetrahydro-1h-cyclopenta[c]pyran-4-carboxylic acid
M391T33	Octyl-3,5-di-tert-butyl-4-hydroxyhydrocinnamate	M563T310	Oleuropein
M411T333_2	4-[(2r,3r,4s,5s,6r)-3,4,5-trihydroxy-6-(hydroxymethyl)oxan-2-yl]oxypentan-2-yl (e)-3-(4-hydroxyphenyl)prop-2-enoate	M573T386	Genipin-gentiobioside
M448T389	Sulprostone	M565T429	Cornuside
M467T336	Cyhalothrin	M497T135	Poricoic acid a
M476T201_2	1-(9z,12z-octadecadienoyl)-2-hydroxy-sn-glycero-3-phosphoethanolamine	M595T249	Ganoderic acid h
M478T201_2	4-[5-[[4-[5-[acetyl(hydroxy)amino]pentylamino]-4-oxobutanoyl]-hydroxyamino]pentylamino]-4-oxobutanoic acid	M601T407	Hydrangenoside c
		M601T33_1	Garcinol
M473T393	Suxibuzone	M619T303_1	(4s,5z,6s)-5-[2-[(e)-3-(4-hydroxy-3-methoxyphenyl)prop-2-enoyl]oxyethylidene]-4-(2-methoxy-2-oxoethyl)-6-[(2s,3r,4s,5s,6r)-3,4,5-trihydroxy-6-(hydroxymethyl)oxan-2-yl]oxy-4h-pyran-3-carboxylic acid
M483T183	(2r,3s,4s,5r,6r)-2-[[(2s,3r,4r)-3,4-dihydroxy-4-(hydroxymethyl)oxolan-2-yl]oxymethyl]-6-[4-(4-hydroxyphenyl)butan-2-yloxy]oxane-3,4,5-triol	M627T376	[(1s,4as,6s,7r,7as)-6-acetyloxy-4-[[(2r,3r,4s,5s,6r)-3-acetyloxy-4,5-dihydroxy-6-(hydroxymethyl)oxan-2-yl]oxymethyl]-7-(acetyloxymethyl)-7-hydroxy-4a,5,6,7a-tetrahydro-1h-cyclopenta[c]pyran-1-yl] 3-methylbutanoate
M524T332	Acrinathrin	M646T402	Aconitine
		M653T419	Bilobalide
M545T185_1	5-heptenoic acid, 7-[(1r,2r,3r,5s)-3,5-dihydroxy-2-[(3r)-3-hydroxy-5-phenylpentyl]cyclopentyl]-, 4-(3-thioxo-3h-1,2-dithiol-5-yl)phenyl ester, (5z)-	M559T37_2	Fahfa 36:3
M574T32	Sulfobacin b	M658T230	Tubeimoside a
M600T39	1,2-dilinoleoylglycerol		
M791T202	Benzoic acid, 3-[(e)-[(3ar,4s,5s,6ar)-4-[(1e,3r)-3-cyclohexyl-3-hydroxy-1-propen-1-yl]hexahydro-5-hydroxy-2(1h)-pentalenylidene]methyl]-, rel-	M659T103	7-(triethylsilyl)-10-deacetylbaccatin iii
M665T38	(2e,4e)-12-[(10e,12e)-13-carboxy-3-hydroxy-	M679T391	Thiofluor 623

	2-(hydroxymethyl)-8,10,12-trimethyltrideca-10,12-dienoyl]oxy-13-(hydroxymethyl)-3,5,7-trimethyltetradeca-2,4-dienedioic acid		
M651T33_1	2,3-dinor-8-isoprostaglandin-f2.alpha.	M757T276	Methyl (2s,3r,4s)-3-ethenyl-4-[(z)-3-[(2s,3r,4r)-3-ethenyl-5-methoxycarbonyl-2-[(2s,3r,4s,5s,6r)-3,4,5-trihydroxy-6-(hydroxymethyl)oxan-2-yl]oxy-3,4-dihydro-2h-pyran-4-yl]-4-oxobut-2-enyl]-2-[(2s,3r,4s,5s,6r)-3,4,5-trihydroxy-6-(hydroxymethyl)oxan-2-yl]oxy-3,4-dihydro-2h-pyran-5-carboxylate
		M816T69_1	Ginsenoside f3
		M829T199_3	Ginsenoside f2
M683T366_5	Isomaltose	M871T33_3	Astragaloside ii
M683T449	Palatinose	M881T374_2	Bussein
M707T77	Prostaglandin e1	M937T137_2	Soyasapogenol b base + o-ddmp, o-hexa-hexa
M725T49_2	N-palmitoyltaurine		
M662T54	Dodecanamide,n-[(1s,2r)-2-hydroxy-1-(hydroxymethyl)heptadecyl]-12-[(7-nitro-2,1,3-benzoxadiazol-4-yl)amino]-	M983T226	[(2r,3s,4s,5r,6s)-6-[(2s,3r,4s,5r,6r)-6-[[(1s,3r,4s,4ar,8ar)-4-[(3s)-3-[(2s,3r,4r,5r,6r)-3,4-dihydroxy-6-methyl-5-[(2s,3r,4r,5r,6s)-3,4,5-trihydroxy-6-methyloxan-2-yl]oxyoxan-2-yl]oxy-3-methylpent-4-enyl]-3,4,8,8a-tetramethyl-1,2,3,4a,5,6-hexahydronaphthalen-1-yl]oxy]-4,5-dihydroxy-2-methyloxan-3-yl]oxy-3,4,5-trihydroxyoxan-2-yl]methyl acetate
M184T69	3,5,9-trioxa-4-phosphatetracosan-1-aminium, 7-(acetyloxy)-24-carboxy-4-hydroxy-n,n,n-trimethyl-, inner salt, 4-oxide, (r)-	M306T36_3	N-acetylsphingosine
M85T85	Valeric acid	M308T36	N-acetyldihydrosphingosine
M922T283	Decanoyl-coa	M513T33_1	N-myristoylsphinganine
M603T204	Morphine n-oxide	M618T35	N-palmitoyl-d-erythro-dihydroceramide-1-phosphate
M488T140	Glycerol tricaprylate	M613T32	Dilinolenin (9c,12c,15c)
M602T594	1-palmitoyl-2-oleoyl-3-linoleoyl-rac-glycerol		
M604T160	1,2-dioleoyl-sn-glycerol	M694T169	Cer 24:1-d7 (d18:1-d7/24:1)
M618T162	1-palmitoyl-2-oleoyl-sn-glycerol	M704T32_1	Palmitoyl sphingomyelin
M104T270_2	Glycerophosphocholine	M1033T42_1	Solasodine base + o-hex-hex-hex-pen
M153T299	Glycerol 3-phosphate	M1035T92	Tomatine
M171T386	Glycerophosphate(2)	M133T154	Estra-4,9-diene-3,17-dione
M789T394_2	N-acetyl-d-lactosamine	M179T35_1	Conessine
M214T393	sn-Glycerol 3-phosphoethanolamine	M287T94_2	Trans-dehydroandrosterone
M313T162	1-Palmitoylglycerol	M309T26	Mestranol
M315T184	2-[2-(3,4-dimethoxyphenyl)ethyl]-4-methoxy-2,3-dihydropyran-6-one	M311T74	21-deoxycortisol
M381T275	1,2-dimyristoyl-sn-glycero-3-phosphate	M329T118	11beta-hydroxyprogesterone
M409T253	1-hexadecanoyl-2-sn-glycero-3-phosphate	M345T208	Corticosterone

M424T206	1-myristoyl-2-hydroxy-sn-glycero-3-phospho ethanolamine	M349T25	Tetrahydrocorticosterone
M454T202	1-palmitoyl-2-hydroxy-sn-glycero-3-phospho ethanolamine	M357T536	.beta.-estradiol 17-valerate
M468T201_2	1-myristoyl-sn-glycero-3-phosphocholine	M367T29_2	3-dehydroepiandrosterone sulfate
M478T168	1-oleoyl-sn-glycero-3-phosphoethanolamine	M381T33	Brassicasterol
M482T199_2	1-pentadecanoyl-sn-glycero-3-phosphocholin e	M383T337	Dehydrocholic acid
M483T159_2	1-palmitoyl-2-hydroxy-sn-glycero-3-phospho -(1'-rac-glycerol)	M395T68	Pregnenolone sulfate
M496T197_2	1-palmitoyl-sn-glycero-3-phosphocholine	M397T42	.beta.-sitosterol
M509T155	1-oleoyl-2-hydroxy-sn-glycero-3-phospho-(1' -rac-glycerol)	M409T137	Beclomethasone
M520T195_2	Lpc 18:2	M413T209	1-hydroxy vitamin d2
M524T193_4	1-stearoyl-2-hydroxy-sn-glycero-3-phosphoc holine	M427T178	Estrone glucuronide
M544T269	1-oleoyl-sn-glycero-3-phosphocholine	M429T33_4	7.alpha.-hydroxy-3-oxo-4-cholestenoic acid
M548T158	1-o-octadecyl-sn-glyceryl-3-phosphorylcholi ne	M431T33_3	3.beta.,7.alpha.-dihydroxy-5-cholestenoic acid
M585T178	1-(1,2-dioctanoylphosphatidyl)inositol	M431T426	Androsterone glucuronide
M647T138_2	1,2-dihexadecanoyl-sn-glycero-3-phosphate	M439T213_2	Spironolactone
M666T240	1-palmitoyl-2-azelaoylphosphatidylcholine	M441T372	Cortisol 21-sulfate
M693T594	[1-hexadecanoyloxy-3-phosphonooxypropan-2-yl]octadec-9-enoate	M443T129	Cinobufagin
M715T37_1	2-linoleoyl-1-palmitoyl-sn-glycero-3-phosph oethanolamine	M443T36_4	Testosterone decanoate
M717T138	1-palmitoyl-3-oleoyl-sn-glycero-2-phosphoet hanolamine	M449T125	5.alpha.-androstan-3.alpha.,17.beta.-diol-o-3-.bet a.-glucuronic acid
M718T223_2	1-palmitoyl-2-(4-ketododec-3-enedioyl)phos phatidylcholine	M465T26	Cholesteryl sulfate
M719T74	Pg 32:1	M467T396	11,17-difuroatemometasone
M722T76	1,2-dipalmitoyl-sn-glycero-3-phospho-(1'-rac -glycerol)	M479T219	Triamcinolone diacetate
M731T150	1,2-dipalmitoleoyl-sn-glycero-3-phosphochol ine	M485T231	Andrastin a
M739T147	Pe 36:4	M485T340	Rutaevin
M739T55	Pe(16:1e/15-hete)	M487T389	Estriol 16alpha-(beta-d-glucuronide)
M741T150	1,2-dilinoleoyl-sn-glycero-3-phosphoethanol amine	M492T73_1	Cevadine
M741T40_1	1-palmitoyl-2-oleoyl-sn-glycero-3-phosphoet hanolamine	M751T113	1-myristoyl-2-palmitoyl-sn-glycero-3-phosphocho line
M743T56_1	1-stearoyl-2-linoleoyl-sn-glycero-3-phosphoe thanolamine	M527T292	.beta.-estradiol-3-glucuronide-17-sulfate
M745T150_1	1,2-dioleoyl-sn-glycero-3-phosphoethanolam	M531T291	Proscillaridin

	ine		
M745T504	1-(1,2r-dioctanoylphosphatidyl)inositol-3,4-bisphosphate	M535T154	Senegenin
M746T73_1	1-palmitoyl-2-linoleoyl-sn-glycero-3-phospho-(1'-rac-glycerol)	M552T243	Histidine conjugated chenodeoxycholic acid
M747T40	1-stearoyl-2-oleoyl-sn-glycero-3-phosphoethanolamine	M579T223	Cucurbitacin e
M784T27	Chenodeoxycholate	M599T464	Methyl 5,6-diacetyloxy-10-hydroxy-2,4b,7,7,10a,12a-hexamethyl-12-methylidene-1,4,8-trioxo-4a,5,6,6a,9,10,10b,11-octahydronaphtho[1,2-h]isochromene-2-carboxylate
M525T28_2	(2s,3r,5r,10r,13r,14s,17s)-2,3,14-trihydroxy-10,13-dimethyl-17-[(2r,3r)-2,3,6-trihydroxy-6-methylheptan-2-yl]-2,3,4,5,9,11,12,15,16,17-decahydro-1h-cyclopenta[a]phenanthren-6-one	M605T107	(5.alpha.)-androstane-3,11,17-trione
M757T148_1	PC(16:0/16:0)	M613T172_2	3-[(cholamidopropyl)dimethylammonio]-1-propanesulfonate
M757T177	1,2-dioleoyl-sn-glycero-3-phosphoethanolamine-n-methyl	M657T227	Fulvestrant 9-sulfone
M759T149_1	1-hexadecanoyl-2-octadecadienoyl-sn-glycero-3-phosphocholine	M699T220	6,8-dihydroxy-2,2,4,4-tetramethyl-7-(3-methylbutanoyl)-9-(2-methylpropyl)-9h-xanthene-1,3-dione
M759T70	1-palmitoyl-2-linoleoyl-sn-glycero-3-phosphocholine	M853T155	.alpha.-chaconine
M770T70_1	1,2-dilinoleoyl-sn-glycero-3-phospho-(1'-rac-glycerol)	M903T40_4	Timosaponin b ii
M772T236	1-palmitoyl-2-oleoyl-phosphatidylglycerol	M545T378	Hypocrellin a
M783T147_1	2-oleoyl-1-palmitoyl-sn-glycero-3-phosphocholine		
M783T196	1-palmitoyl-2-arachidonoyl-sn-glycero-3-phosphoserine	M428T430_4	Adenosine 5'-diphosphate
M664T434	Nicotinamide adenine dinucleotide (NAD)	M444T430	Adenosine 5'-phosphosulfate
M298T98	S-methyl-5'-thioadenosine	M542T434	ADP-ribose
M383T391	S-adenosyl-l-homocysteine	M588T431	GDP-L-Fucose
M398T161	S-Adenosylmethionine		
M457T393_1	Flavin mononucleotide (fmn)	M939T425	Bucladesine
M337T408	5'-phosphoribosyl-5-amino-4-imidazolecarboxamide (aicar)	M336T465	Nicotinate d-ribonucleotide
M243T160	Pseudouridine	M126T190	5-methyl-2'-deoxycytidine
M257T85	2'-o-methyluridine	M211T84	2,3-dideoxyuridine
M341T163	(+)-pinoresinol	M225T497	His-ser
M380T34_1	Benthiavalicarb-isopropyl	M227T112	Ile-Pro
M152T360	Deoxyguanosine	M243T437	Uridine
M252T142	Deoxyadenosine	M255T53	5-ethyl-2'-deoxyuridine

M253T184	Deoxyinosine	M487T240	Cytidine
M267T34	Inosine	M245T442	Cytidine 5'-diphosphocholine
M268T171	Adenosine	M267T33	Stearaldehyde
M282T105	2'-o-methyladenosine	M323T441	Uridine 5'-monophosphate
M282T353	Guanosine	M324T458	Cytidine 5'-monophosphate
M297T210	1-methylguanosine	M325T441	Uridine 5'-monophosphate (UMP)
M595T417	7-methylguanosine	M369T242	Deoxyuridine monophosphate (dUMP)
M158T306	Acetyl-DL-Valine	M403T431	Uridine 5'-diphosphate (UDP)
M203T560	NG,NG-dimethyl-L-arginine(ADMA)	M403T473	Uridine 5'-diphosphate
M335T220	Strychnine	M489T442	Cytidine 5'-diphosphocholine (CDP-choline)
M348T331	Adenosine monophosphate (AMP)	M606T432	UDP-N-acetylglucosamine
M348T430	Adenosine 5'-monophosphate	M370T280	Adenosine 3'-monophosphate
M362T191	Guanosine 5'-monophosphate		
M100T331	3-Butynoic acid	M239T32_1	Cystine
M100T57	5-aminovaleric acid	M240T394	S-(4-fluorophenyl)mercapturic acid
M102T299	4-hydroxy-l-isoleucine	M242T367	Pro-gln
M102T374	Dimethylglycine	M243T353	Glu-Pro
M102T78	Stachydrine	M244T251	Agomelatine
M103T396	Malonic acid	M244T267	Ala-Arg
M104T373	4-Aminobutyric acid(GABA)	M245T187_2	Leu-Leu
M106T417	Serine	M245T232	Palythine
M110T454	N-.alpha.-(tert-butoxycarbonyl)-l-histidine	M245T379	gamma-L-Glutamyl-L-valine
M112T85	Leucenol	M246T487	Arg-Ala
M114T271	L-Proline	M246T535	Lys-Val
M114T305	Hydroxyproline	M247T240_2	Lenticin
M114T311_2	Proline	M249T352	Glu-Thr
M114T349	D-Proline	M249T463	Asp-Asp
M114T55	Maleamic acid	M251T310	His-Pro
M115T52	Maleic acid	M252T48_2	Acetyl Tyrosine Ethyl Ester
M116T341	DL-proline	M253T407	D-galacturonic acid
M116T521	L-hydroxyarginine	M253T42	N-(2,4-dinitrophenyl)-l-alanine methyl ester
M117T390_2	Succinate	M260T58	Gly-Trp
M118T116	Guanidoacetic acid	M261T459	Asp-Glu
M118T153_1	L-homoserine	M263T69	Pro-phe
M118T303_2	DL-valine	M267T214	Phe-thr
M118T431	Dl-homocysteine	M268T330	N-Acetyl-L-tyrosine
M119T520	Tartronate	M269T298	His-Leu
M124T241	1-methyl-l-histidine	M269T301	Dflnha
M128T228	4-Guanidinobutyric acid	M275T369	Gln-gln
M128T302_2	L-pyroglutamic acid	M275T461_2	Gamma-l-glutamyl-l-glutamic acid
M130T269_2	Isoleucine	M277T157	Stearidonic Acid
M130T282	L-arginine, methyl ester	M277T172	Leu-Phe
M130T375	D-pyroglutamic acid	M277T370	Gamma-glutamylmethionine
M130T382	N-Acetyl-L-alanine	M277T38	all cis-(6,9,12)-Linolenic acid

M131T201	Asparagine	M277T450	L-Saccharopine
M131T241	DL-3-Phenyllactic acid	M279T224	Val-Tyr
M131T374	Glutaric acid	M283T426	Glu-His
M132T354	L-Isoleucine	M284T191	His-Lys
M132T405	D-aspartic acid	M284T212	Oxymorphone
M132T53	Cis-4-hydroxy-l-proline	M286T418	Hydromorphone-3-.beta.-d-glucuronide
M132T91_2	Ser-Arg	M288T399	Ophthalmate
M134T234	N-methyl-l-threonine	M290T344	N-fructosyl pyroglutamate
M134T404	L-aspartic acid	M293T26	Cinchonine
M134T62	O-t-butyl-l-threonine methyl ester	M297T175	Met-Phe
M136T430	S-Methyl-L-cysteine	M300T208	Ile-Gly-Ile
M140T67	3-amino-2,3-dihydrobenzoic acid	M302T420	Gly-Pro-Glu
M143T338	Ectoine	M303T413	Arg-Gln
M144T258	Dl-2-methylglutamic acid	M304T489	Arg-glu
M144T261	N.alpha.-methyl-l-lysine	M306T567	L-glutathione, reduced
M144T377	Isobutyrylglycine	M307T265	Cys-Gly-Lys
M144T60	L-homoarginine	M308T403	Glutathione
M144T96	L-glutamic acid, dimethyl ester	M309T351	Gamma-glu-tyr
M145T375	Glutamine	M318T186	Trp-Ile
M146T237	N-methyl-l-isoleucine	M318T456	Tyr-His
M146T261	DL-O-tyrosine	M323T257_2	Alpha-N-Phenylacetyl-L-glutamine
M385T194	2-[5-[2-[2-[5-(2-hydroxypropyl)oxolan-2-yl] propanoyloxy]propyl]oxolan-2-yl]propanoic acid	M325T37	[(4e)-7-acetyloxy-6-hydroxy-2-methyl-10-oxo-2, 3,6,7,8,9-hexahydrooxecin-3-yl] (e)-but-2-enoate
M147T254_2	D-glutamine	M328T329	Arg-Ser
M147T276_1	L-Arabinono-1,4-lactone	M329T171	Tyr-Met(o)
M147T430	DL-glutamine	M334T265	Trp-Met
M147T521	DL-Lysine	M337T36_2	MG(18:2(9Z,12Z)/0:0/0:0)[rac]
M148T344	L-Methionine	M338T168	Tazarotenic acid sulfoxide
M148T397_2	Glutamic acid	M338T268	Met-Gln
M151T384_1	Cysteic acid	M339T27	Gly-His-Lys
M151T65	Pro-Gly-Lys	M339T35_1	Dl-sulforaphane n-acetyl-l-cysteine
M152T287	Gln-arg	M347T580	L-Arginine
M153T262	N2-trifluoroacetyl-l-glutamine	M349T382	N-Acetyl-L-aspartic acid
M156T302	L-Histidine	M351T457	Cys-Thr-Lys
M156T403	Histidine	M357T400_1	Pyroglu-Ala-Arg
M157T183	L-dihydroorotate	M359T53	Nicotinuric acid
M158T300	Diethyl l-glutamate	M360T234	N-arachidonoyl-l-serine
M158T405	N.alpha.-(tert-butoxycarbonyl)-l-aspartic aci d	M366T445	Trp(dioxidation)-Glu
M159T269	Gluconolactone	M371T543	Pyroglu-Ile-Lys
M162T299	Acetylcysteine	M373T541	Val-Val-Arg
M162T35	4-hydroxy-l-glutamic acid	M377T59	Neotame
M162T409	2-aminoadipic acid	M380T185_1	S-lactoylglutathione

M164T268	Phenylalanine	M381T27_4	L-alaninamide, n-[2-[2-(hydroxyamino)-2-oxoethyl]-4-methyl-1-oxopentyl]-3-methyl-l-valyl-n-(2-aminoethyl)-
M165T107	1,2-Benzenedicarboxylic acid	M391T392	1,3-Dimethyluric acid
M146T302	N-acetylserine	M402T437	Tyr-Gly-Tyr
M165T194	Atrolactic acid	M403T392_2	Asp-Leu-Arg
M166T260	DL-phenylalanine	M405T306	1h-indazole-3-carboxamide, n-[(1s)-2-amino-2-oxo-1-(phenylmethyl)ethyl]-1-(cyclohexylmethyl)-
M168T356_1	1-methylhistidine	M417T505	Arg-Asn-Lys
M168T384	4-hydroxy-l-phenylglycine	M418T169	Philanthotoxin 74
M635T492	Glutathione disulfide	M431T307	Captopril disulfide
M170T416	3-Methylhistidine	M437T276	Pantothenic acid
M171T233	Pro-Pro-Lys		
M172T332	L-leucine, n-acetyl	M439T58	Quinapril
M172T43	Gabapentin		
M173T375	Isocitric acid	M444T176	Dihydrofolic acid
M173T441	Cis-aconitate	M446T441	Tetrahydrofolate
M174T275	L-citrulline	M449T33	N-acetyl-p-fluoro-dl-phenylalanine
M174T392	Guanidinosuccinic acid	M459T257	Raltitrexed
M174T473	Ala-Thr-Arg	M459T408_2	Ergothioneine
M175T282_2	N-.alpha.-acetyl-l-ornithine	M472T278	Folinic acid
M175T395	Arginine	M475T219_2	Asn-Trp-Arg
M175T429	Ser-Ser-Arg	M475T34	Thyroxine 4'-o-.beta.-d-glucuronide
M175T521_2	DL-arginine	M484T460	Karakin
M177T351	L-Ascorbic acid	M487T250	N-(aminothioxomethyl)-5-oxo-1-propyl-2-pyrrolidineacetamide
M177T401	3-Hydroxy-4-methoxycinnamic acid	M492T472	Trp-Met-Arg
M180T497	Ser-Pro-Arg	M497T336	Chicoric acid
M182T158	N-acetyl-o-fluoro-dl-phenylalanine	M533T294	S-(2,3,4-trihydroxybutyl)mercapturic acid
M182T303_2	DL-tyrosine	M542T417	Phosphoramidon
M185T140	Gabapentin related compound e	M590T537	Benzoic acid, 3-[[(3-carboxycyclohexyl)amino]carbonyl]-4-[3-[4-(4-phenoxybutoxy)phenyl]propoxy]-
M185T50	Fuberidazole	M593T183	Pantethine
M187T111	Nepsilon,nepsilon,nepsilon-trimethyllysine	M607T349	Melphalan
M597T250	Phe-met-arg-phe-amide	M613T493	Glutathione, oxidized
M185T58_2	3-Phosphoserine	M627T173_2	1(2h)-pyrimidineacetamide, n-[(1s,3s,4s)-4-[[2-(2,6-dimethylphenoxy)acetyl]amino]-3-hydroxy-5-phenyl-1-(phenylmethyl)pentyl]tetrahydro-4-hydroxy-.alpha.-(1-methylethyl)-2-oxo-, (.alpha.s)-
M187T265	Val-Ala	M663T406	Zanamivir
M187T302	Pro-Ala	M671T73	1-Palmitoyl-2-linoleoyl-sn-glycero-3-phosphate
M187T375	Homocitrate	M683T350	(1s,3r,4s,5r)-4-[(4-carboxy-3-hydroxy-3-methylbutanoyl)oxy]-3,5-bis({[(2e)-3-(3,4-dihydroxypheny

			l)prop-2-enoyl]oxy})-1-hydroxycyclohexane-1-carboxylic acid
M187T401	N-acetylglutamine	M189T537	N6,N6,N6-Trimethyl-L-lysine
M629T172_1	Lopinavir	M735T393_1	(phenoxymethyl)penicilloic acid
M188T383	N-acetyl-l-glutamate		
M189T234	Thr-Thr-Arg	M787T394	Phomopsin a
M189T517	L-ng-monomethylarginine		
M729T391	Glycine, n,n'-[1,2-ethanediylbis(oxy-2,1-phenylene)]bis[n-[2-[(acetyloxy)methoxy]-2-oxoethyl]-, 1,1'-bis[(acetyloxy)methyl] ester	M84T375	1-Aminocyclopropanecarboxylic acid
M189T57	Glycyl-l-4-hydroxyproline	M88T348	Sarcosine
M190T344	N-acetyl-l-methionine	M90T347	Alanine
M190T381_2	.alpha.-guanidinoglutaric acid		
M190T50	D-4-Hydroxyphenylglycine	M90T418	L-Alanine
M191T33	Isoscopoletin		
M191T422	Ureidoglutaric acid		
M191T468	Citrate	M92T296	Alanine-2,3,3,3-d4
M192T267	Thioarginine	M932T39	Angiotensin iii
M194T212	2-methyl-n-(4-methylphenyl)alanine	M99T430	2-hydroxyethyl acrylate
M194T334	Pro-Asp-Arg	M103T237	Beta-hydroxybutyrate
M195T68	Leu-Thr-Arg	M103T395	3-hydroxybutyric acid
M198T107	Kinetin	M129T180	L-2-hydroxyglutaric acid
M199T222_2	O-Phospho-L-homoserine	M131T129_2	6-hydroxyhexanoate
M200T114	N5-(1-imino-3-butenyl)-l-ornithine	M133T405_2	Malate
M201T262	Pro-Glu-Arg	M133T576	L-Malic acid
M201T529	Ile-Leu-Arg	M147T267	3-hydroxyglutaric acid
M202T70	Pro-Met-Arg	M147T435	(S)-2-Hydroxyglutarate
M203T229	Pro-ser	M159T131	3-hydroxyoctanoic acid
M203T374	Glu-Thr-Arg	M167T29	p-Hydroxymandelic acid
M203T504_2	Ng,ng-dimethyl-l-arginine	M179T159	(+)-dimethyl l-tartrate
M203T65	.alpha.-L-Glu-Gly	M203T292	3,5-dihydroxydecanoic acid
M204T445	Ala-Asn	M215T216	3-hydroxydodecanoic acid
M206T302	S-methyl-l-thiocitrulline	M89T269	Dl-lactate
M206T322	N-Acetyl-L-phenylalanine	M89T307	.beta.-hydroxypropionic acid
M209T298	Dl-lanthionine	M115T208	Alpha-ketoisovaleric acid
M210T71	Glu-Asp-Arg	M129T186	Dihydroxyfumarate
M213T107	m-Chlorohippuric acid	M129T294	2-ketohexanoic acid
M213T419	Pro-pro	M129T341	4-ketopimelic acid
M214T175	L-n5-(1-imino-3-pentenyl)ornithine	M157T280_2	2-oxooctanoic acid
M215T292	Pro-val	M159T137_2	2-oxoadipic acid
M215T49	7-aminodesacetoxycephalosporanic acid	M87T280	Pyruvate
M216T343	Gln-Ala	M123T267	Ethyl hydrogen methylphosphonate
M217T216	Val-Val	M137T461	Phosphomycin
M217T371	Glu-Glu-Arg	M555T83	Tetradecylphosphonate

M218T72	Captopril	M153T283	O,o-diethyl phosphate
M219T278_1	Phe-Asp-Arg	M211T221	(s)-dibutyl 3-hydroxybutyl phosphate
M219T418	5-L-Glutamyl-L-alanine	M237T29_2	D-erythro-imidazolylglycerol phosphate
M220T359	N.omega.-nitro-l-arginine	M592T398_1	Creatinol o-phosphate
M222T392	His-Met-Arg	M737T424	Tritolyl phosphate
M224T241_1	Dacarbazine	M124T294	Taurine
M227T169	Trifluoroacetic acid	M180T32	Acamprosate
M227T171	Nitrotyrosine	M181T158_2	(n-(-2-acetamido))-2-aminoethanesulfonic acid
M227T185	Butyric acid, 4-[(4-chloro-o-tolyl)oxy]-	M487T13	N-[tris(hydroxymethyl)methyl]-3-aminopropanesulfonic acid
M227T341	His-Ala	M239T189	.beta.-carboline-1-propionic acid
M229T304_1	Pro-leu	M209T27	N-octyl sulfate
M229T424	Pro-hyp	M1150T140_1	Valinomycin
M229T549	L-leucyl-l-proline	M156T454	L-carnosine
M230T43_1	Arg-Gln-Arg	M473T27	Amastatin
M231T200	Dl-Leu-dl-Val	M657T38	Enniatin b
M231T402_2	N-Acetyl-L-glutamic acid	M671T38	(3s,6r,9s,12r,15s,18r)-3-butan-2-yl-4,10,16-trimethyl-6,9,12,15,18-penta(propan-2-yl)-1,7,13-trioxa-4,10,16-triazacyclooctadecane-2,5,8,11,14,17-hexone
M232T382	Arg-gly	M690T238	Enniatin a1
M517T279	N-[tris(hydroxymethyl)methyl]-3-amino-2-hydroxypropanesulfonic acid	M709T293	Jasplakinolide
M233T401	Pyroglu-Cys	M754T186	Microcolin b
M234T245_2	L-ng-nitroarginine methyl ester	M178T66	Cyclohexanesulfamic acid
M235T430	Glu-Ser	M236T30_2	3-(cyclohexylamino)-2-hydroxy-1-propanesulfonic acid
M238T107	N-(2,4-dinitrophenyl)-l-valine	M258T142	Myristamine oxide
M125T180	Nitrosobenzene	M273T44	Thiofanox sulfone
M112T392	Histamine	M282T173	Sphingosine
M146T194	Acetylcholine	M288T40_3	Heptadecasphinganine
M160T386	Cyclohexylamine	M300T126	Phytosphingosine
M162T355	L-carnitine	M392T214_1	Guanidinopropionic acid
M184T386	Phosphorylcholine		
M199T428	Cymoxanil	M605T225	Chlorambucil
M210T166	2,2-bis[hydroxymethyl]-2,2',2"-nitrilotriethanol	M60T271	Trimethylamine
M211T350_2	N-(4-chlorophenyl)-4-piperidinamine	M72T303	1,2-diamino-2-methylpropane
M213T382	Triethanolamine	M785T63	Arachidonoylthiophosphorylcholine
M214T103	Tetradecylamine	M253T497	1,2,3-Benzenetriol
M225T350	Porphobilinogen	M327T357	L-Fucose
M127T366_2	1,6-anhydro-2,3-o-isopropylidene-.beta.-d-mannopyranose	M328T295	Lithosprmoside

M193T327	Perseitol	M328T399	P-acetamidophenyl .beta.-d-glucuronide
M193T35_1	Myristicine	M341T366_11	Sucrose
M390T108	Retrorsine n-oxide	M341T398	Trehalose
M1043T362_2	Lstc	M341T461	Galactinol
M105T305	Glyceric acid	M347T450	Acetohexamide
M1081T284	Blood group a trisaccharide	M359T324	D-fructose
M109T269	Quinone	M360T153	Coniferin
M111T469_2	Dihydroxyacetone	M360T398	Melibiose
M121T37	4-hydroxybenzaldehyde	M363T29	3,6,9,12-tetraoxatetracosan-1-ol
M132T135	S(-)-cathinone	M366T32_1	2(1h)-pyridinone, 3-[(2s,4s,5r)-5,6-dichloro-2,4-dimethyl-1-oxohexyl]-4-hydroxy-5,6-dimethoxy-
M137T341_2	Shikimate	M367T401	2-[1-hydroxy-1-(4-methoxyphenyl)propan-2-yl]oxy-6-(hydroxymethyl)oxane-3,4,5-triol
M133T113	2'-Deoxy-D-ribose	M382T264_1	Cis-zeatin-9-glucoside
M365T497	Nigerose	M389T381_1	Gilvocarcin v
M401T263	Acetic acid, 2-[4-[3-(4-acetyl-3-hydroxy-2-propylphenoxy)propoxy]phenoxy]-	M390T157_1	Syringin
M143T43	Pyruvaldehyde	M141T242	(-)-quebrachitol
M145T237_1	L-Rhamnose	M401T366_2	Gentiopicroside
M149T151	D-ribose	M407T27_2	Repaglinide acyl-.beta.-d-glucuronide
M149T348	D-lyxose	M413T266	Bispyribac
M151T171	D-xylose	M415T429_2	(2r,3s,4s,5r,6r)-2-[[(2s,3r,4r)-3,4-dihydroxy-4-(hydroxymethyl)oxolan-2-yl]oxymethyl]-6-(2-phenylethoxy)oxane-3,4,5-triol
M151T240_2	Ribitol	M421T476	.alpha.,.alpha.'-trehalose 6-phosphate
M151T27	Triethylene glycol	M431T342	Osmanthuside h
M439T428	(2r,3s,4s,5r,6r)-5-[(2s,3r,4r)-3,4-dihydroxy-4-(hydroxymethyl)oxolan-2-yl]oxy-2-(hydroxymethyl)-6-(2-phenylethoxy)oxane-3,4-diol	M432T164_1	Trifluperidol
M155T38	4-hydroperoxy-2-nonenal		
M162T121	D-mannosamine		
M163T201	Dulcitol	M433T34_1	Chaetoviridin a
M163T305	D-quinovose	M151T302	Xylitol
M165T282	L-fucitol	M447T348	N,n'-diacetylchitobiose
M165T359	D-arabinonic acid	M453T135	Oleanonic acid
M169T31	Dihydroxyacetone phosphate	M465T412	Catalposide
M177T107	D-glucono-1,5-lactone	M483T108	Octapropylene glycol
M179T308	Alpha-D-Glucose	M483T418	Paeonolide
M179T335	L-Sorbose	M485T410	Disialyllactose
M179T366	D-psicose	M487T373	Blood group b trisaccharide
M179T392	Myo-inositol	M487T451	3.alpha.,6.alpha.-mannotriose
M181T275	D-sorbitol	M497T403	Verproside

M183T408	D-Mannitol	M498T304	Lsta
M186T105	N-Acetylmannosamine	M499T284	Isochlorogenic acid b
M191T268	Quinate	M503T449_2	Raffinose
M191T279	D-glucarate	M503T484	Maltotetraose
M191T358	Mucic acid	M508T381_1	(methylsulfonyl)octyl glucosinolate
M193T235	2-keto-l-gulonic acid	M509T450	3'-galactosyllactose
M193T268_1	D-pinitol	M511T25	2'-fucosyllactose
M193T35_2	Tripropylene glycol	M515T205_1	Cynarin
M193T357	D-glucuronic acid	M519T427	D-glucosamine 1-phosphate
M195T343	Resodiacetophenone	M522T450_3	Maltotriose
M195T453	D-gluconate	M525T345_1	10,10'-oxybis(phenoxarsine)
M195T550	L-Threonate	M527T260	Tremulacin
M198T366	D-Mannose	M527T329	3'-fucosyllactose
M209T437	D-Galactarate	M527T450_4	Melezitose
M211T293	Adenosine 2'-monophosphate	M540T434	Cyclic adenosine diphosphate ribose
M212T379	Methyl .alpha.-d-mannopyranoside	M549T446	Gentianose
M218T276	Pantothenate	M564T378_1	Thiocolchicoside
M220T222	N-acetyl-d-glucosamine	M576T282	Cis-moxifloxacin acyl-.beta.-d-glucuronide
M222T262	N-acetyl-d-galactosaminitol	M601T430	N-acetyl-d-glucosamine 6-phosphate
M222T368	N-acetyl-d-mannosamine	M614T229	Hexosyl lpe 16:0
M225T476	D-Mannose-6-phosphate	M623T434	N-acetylneuraminic acid dimer
M229T106	D-ribulose 5-phosphate	M632T394	6'-sialyllactose
M229T403	D-ribose 1-phosphate	M643T414	Neohesperidose heptaacetate
M237T369	2-keto-3-deoxyoctonic acid	M649T465	N-glycolylneuraminic acid
M239T244	Curvulin	M653T347	Leiocarposide
M240T338	D-glucosamine 6-phosphate	M665T496_2	Stachyose
M241T288	.alpha.-d-galactose 1-phosphate	M666T170	Cer 22:1-d7 (d18:1-d7/22:1)
M241T399	Glucose 1-phosphate	M674T286	Lewis b tetrasaccharide
M243T456	L-fucose-1-phosphate	M675T374	Blood group h disaccharide
M258T418	Ile-Lys	M676T371	Lewis y tetrasaccharide
M259T185	Fructose 1-phosphate	M679T420	3'-.alpha.-sialyl-n-acetyllactosamine
M259T430	D-fructose 6-phosphate	M679T67	Dipyridamole mono-o-.beta.-d-glucuronide
M259T485	alpha-D-Galactose 1-phosphate	M680T399	Acarbose
M261T197	Sorbitol 6-phosphate	M705T497	Laminaritetraose
M261T464	D-mannitol 1-phosphate	M707T365	4.alpha.-mannobiose
M273T453	1-Deoxy-D-xylulose 5-phosphate	M746T436	Lnnt
M281T372_2	Ethyl glucuronide	M755T380_1	Forsythoside b
M289T464	D-Ribose 5-phosphate	M785T502	Flavin adenine dinucleotide (FAD)
M293T63	Embelin	M809T241_2	Echinacoside
M297T471	D-Mannose 1-phosphate	M819T23	Aloenin
M299T341	Benzoic acid + 1o, o-hex	M819T412	B-pentasaccharide
M300T444	N-Acetylglucosamine 1-phosphate	M827T511_2	Pentasaccharides (hex-hex-hex-hex-hex)
M315T249	Benzoic acid + 2o, o-hex	M83T300	Glutaraldehyde
M317T33_1	1,4-d-xylobiose	M846T512_1	Maltopentaose

		0	
M317T80	Arbutin	M865T422	Sialyl lewis x
M319T344	Propofol .beta.-d-glucuronide	M887T297_2	[(2r,3r,4r,5r,6r)-2-[[(2r,3r,4r)-3,4-dihydroxy-4-(hydroxymethyl)oxolan-2-yl]oxymethyl]-4-[(2s,3r,4r,5r,6s)-4,5-dihydroxy-6-methyl-3-[(2s,3r,4s,5s)-3,4,5-trihydroxyoxan-2-yl]oxyoxan-2-yl]oxy-6-[2-(3,4-dihydroxyphenyl)ethoxy]-5-hydroxyoxan-3-yl] (e)-3-(3,4-dihydroxyphenyl)prop-2-enoate
M323T348	D-myo-inositol-1,3-diphosphate	M911T170	Man3 a
M295T544	Propanoic acid, 3-[[[2-[(aminoiminomethyl)amino]-4-thiazolyl]methyl]thio]-	M913T304	G2f
M311T497	1h-pyrrole-3-propanoic acid, 5-[(1,2-dihydro-2-oxo-3h-indol-3-ylidene)methyl]-2,4-dimethyl-	M283T29	Tropisetron
M324T28_2	Cycloxydime	M203T261	Tryptophan
M325T451	Cellobiose	M204T265	L-tryptophanamide
M326T322	Hboa + o-hex	M204T461	5-methoxyindoleacetate
M100T356	Hymexazole	M217T252	Carboline base + 4h, carboxylic acid
M121T182_2	4-imidazoleacrylic acid	M221T282_1	Indole-3-pyruvic acid
M139T182	Urocanate	M278T156_1	Indirubin-3'-monoxime
M142T392	Dimetridazole	M550T191	1-O-(cis-9-Octadecenyl)-2-O-acetyl-sn-glycero-3-phosphocholine
M159T184_1	Allantoin	M312T245	Indomethacin
M235T142	Zolpidem	M323T283	3-pyridinecarboxamide, n-(6-chloro-9h-pyrido[3,4-b]indol-8-yl)-
M315T81	Febuxostat	M357T138	Eseroline fumarate
M385T35_3	Propylpyrazoletriol		
M415T350	Pyraflufen-ethyl	M395T464	1-(4-fluorobenzyl)-n-(naphthalen-1-yl)-1h-indole-3-carboxamide
M430T401_1	Tubacin		
M85T263	1h-1,2,4-triazol-3-amine	M464T219	Benzamide, n-[5-[2-(3,5-dimethoxyphenyl)ethyl]-1h-pyrazol-3-yl]-4-[(3r,5s)-3,5-dimethyl-1-piperazinyl]-, rel-
M620T232	N-[(2s)-2-[[(1z)-1-methyl-3-oxo-3-[4-(trifluoromethyl)phenyl]-1-propen-1-yl]amino]-3-[4-[2-(5-methyl-2-phenyl-4-oxazolyl)ethoxy]phenyl]propyl]propanamide	M399T33_4	Methanone, [6-hydroxy-1-[2-(4-morpholinyl)ethyl]-1h-indol-3-yl]-1-naphthalenyl-
M586T378	.beta.-d-glucopyranosiduronic acid, 6-[3-(1-naphthalenylcarbonyl)-1h-indol-1-yl]hexyl	M407T259	DL-tryptophan
M251T417	Phenytoin	M413T302_2	Bisindolylmaleimide i
M487T346	2,4-imidazolidinedione, 1,3-bis(4-bromophenyl)-5-phenyl-	M439T294	5-hydroxy-l-tryptophan
M281T142	Cyclopeptine	M441T36_3	Oleoylserotonin
M301T464	Temazepam	M500T77_2	.beta.-d-glucopyranosiduronic acid, 4-[3-(1-naphthalenylcarbonyl)-1h-indol-1-yl]butyl

M572T414	.beta.-d-glucopyranosiduronic acid, 5-[3-(1-naphthalenylcarbonyl)-1h-indol-1-yl]pentyl	M571T280	Benzoylstaurosporine
M352T234	Pirenzepine	M302T83_2	Noroxycodone
M161T194	Mellein	M583T482	Stauprimide
M299T176	Amlexanox	M130T268	Goitrin
M379T375	8-desoxygartanin	M669T338	Becatecarin
M395T272	Gartanin	M556T403	Lithospermic acid
M257T166	1-Methylpseudouridine	M641T421	(3z)-5-[(2,6-dichlorobenzyl)sulfonyl]-3-[(3,5-dimethyl-4-{[(2r)-2-(1-pyrrolidinylmethyl)-1-pyrrolidinyl]carbonyl}-1h-pyrrol-2-yl)methylene]-1,3-dihydro-2h-indol-2-one
M385T29	6h-dibenzo[b,d]pyran, 3-(1,1-dimethylheptyl)-6a,7,10,10a-tetrahydro-1-methoxy-6,6,9-trimethyl-, (6ar,10ar)-	M907T231	Antibiotic k 252b
M409T167	Mangostine	M358T414	1h-indazole-1-pentanoic acid, 3-[[[1-(aminocarbonyl)-2,2-dimethylpropyl]amino]carbonyl]-
M423T48	Spiro[isobenzofuran-1(3h),9'-[9h]xanthen]-3-one, 3'-hydroxy-6'-(4-hydroxyphenoxy)-	M227T79	.epsilon.-caprolactam
M431T242	Khelloside	M314T226	Ampicillin
M435T336_2	Irisxanthone	M362T108	Cefadroxil
M449T204	Cratoxyarborenone e	M390T390	Cefaclor
M453T396	4'-o-.beta.-d-glucosyl-5-o-methylvisamminol	M423T136	Cefuroxime
M473T342	Garcinone c	M455T347	Cefazolin
M583T493	Neomangiferin	M127T375	Dihydro-4,4-dimethyl-2,3-furandione
M637T39	Decahydrogambogic acid	M195T379	L-gulono-1,4-lactone
M693T308	Acetyl isogambogic acid	M237T229_1	L-Gulonic gamma-lactone
M625T304	Dauricine	M507T27_2	3-furancarboxylic acid, tetrahydro-4-methylene-2-octyl-5-oxo-, (2r,3s)-
M438T215	2-(r)-[1-(r)-(3,5-bis(trifluoromethyl)phenyl)ethoxy]-3-(s)-fluorophenylmorpholine	M404T353	Perphenazine
M452T33_1	7-hydroxyfluphenazine	M557T295	Aprepitant
M296T48	Dothiepin	M116T207	N-methyl-n-(tetrahydro-2-furanylmethyl)-4-piperidinamine
M255T44	Cinnavalininate	M128T73	2-(4-amino-1-piperidinyl)ethanol
M187T265_2	Deoxyvasicinone	M143T264	1-acetyl-3-piperidinamine
M189T278	Vasicine	M330T413	Paroxetine
M203T282_1	Vasicinone	M423T179	3'-hydroxyrepaglinide
M475T106	Vandetanib	M386T337	Methanone, (4-methoxy-1-naphthalenyl)(2-methyl-1-pentyl-1h-indol-3-yl)-
M695T420	Benzenesulfonamide, 2-methoxy-n-(1,2,3,4-tetrahydro-3-methyl-2-oxo-6-quinazolinyl)-	M252T66	D-neopterin
M260T559	Diethylcarbamazine	M325T224	Methopterine
M303T282	1,4-piperazinediethanesulfonic acid	M325T328	Pterine
M450T394	Aripiprazole	M359T389	(-)-riboflavin

M243T62	Lumichrome	M455T429	Methotrexate
M470T415	Nefazodone	M483T228_2	Tetrahydro-l-biopterin
M531T306	Ketoconazole	M125T268	Triacetic acid lactone
M723T304	Hydroxyitraconazole	M859T388	Fostriecin
M109T358	Dihydrothymine	M122T359	Quinolinate
M112T196	Cytosine	M123T64	Niacinamide
M113T161	Uracil	M124T341_2	Nicotinate
M154T60	Methyl 3-aminopyrazine-2-carboxylic acid	M163T77	Nicotine
M183T248	Barbital	M166T235	Pyridoxal
M225T35	5-acetylamino-6-formylamino-3-methyluracil	M168T236	Pyridoxal (Vitamin B6)
M265T348	Thiamine	M170T108	Pyridoxine
M343T63	Thiamine monophosphate	M170T82	1,5-naphthyridine, 2-[3-(6-methyl-2-pyridinyl)-1 h-pyrazol-4-yl]-
M379T243	Pyridate	M180T234	Fusaric acid
M559T389	Cidofovir	M182T46	4-pyridoxic acid
M574T381_1	Bosentan	M229T266	Aspernigrin a
M115T331	Vitamin c	M241T306_1	Pheniramine
M111T331	2-furancarboxylic acid	M316T289	Furametpyr
M323T31_3	5-(tetradecyloxy)-2-furoic acid	M454T339	1-butanol, 2-[[9-(1-methylethyl)-6-[[[4-(2-pyridin yl)phenyl]methyl]amino]-9h-purin-2-yl]amino]-, (2r)-
M811T216	4-thiazolidinone, 5-[[1-(2,4-difluorophenyl)-2,5-dimethyl-1h-pyrrol-3-yl]methylene]-2-[(2-methoxyethyl)imino]-3-methyl-, (2z,5z)-	M495T427	Pyridoxamine 5-phosphate
M129T137_2	Dehydro-l-(+)-ascorbic acid dimer	M613T419	Herbimycin a
M351T490	D-Glucuronolactone	M70T311_2	2-Amino-2-methyl-1,3-propanediol
M136T170	Adenine	M94T85	3-hydroxypyridine
M137T217	Hypoxanthine	M95T392	3-aminopyridine
M150T105	7-methyladenine	M96T54	2(1h)-pyridinone
M150T241	3-methyladenine	M110T160	Pyrrole-2-carboxylic acid
M151T216	Xanthine	M541T165_2	Atorvastatin
M152T263	2-Hydroxyadenine	M339T244	Alpha-guaiaconic acid
M166T112	7-Methylxanthine	M114T50	Ethosuximide
M166T323	7-methylguanine	M219T161	Carphedon
M180T286_2	2-dimethylamino-6-hydroxypurine	M72T277_2	Pyrrolidine
M193T304	Caffeine	M144T86	Quinolin-2-ol
M213T28	8-chlorotheophylline	M145T184	3-aminoquinoline
M363T33	1-methyluric acid	M146T67	4-hydroxyquinoline
M451T348	Acyclovir	M159T117	2-heptyl-4-hydroxyquinoline n-oxide
M575T381	Tenofovir	M162T104	8-fluoroquinolin-4-ol
M205T32	Levamisole	M172T356	Quinaldic acid
M118T261	Indole	M172T52_2	8-hydroxyquinoline-2-carbaldehyde
M130T54	Indoleacetic acid	M174T183	4-quinolinecarboxylate
M132T48	3-Methylindole	M188T185	Kynurenic acid

M142T200	Indoleacrylic acid	M204T33	Xanthurenic acid
M144T201	Ala-Ala	M322T497	5,8-quinolinedione, 6-chloro-7-[[2-(4-morpholinyl)ethyl]amino]-
M144T36	Indole-3-carboxaldehyde	M553T180	4-(2-oxo-1,2,3,4-tetrahydroquinolin-6-yl)oxycilostazol
M157T136	Indole-3-acetonitrile	M579T165	Hematoporphyrin
M158T353	Indole-3-butyric acid	M593T240	Pheophorbide a
M160T33	Indole-3-carboxylic acid	M655T456	Coproporphyrin I
M160T47	Indole-3-acetaldehyde	M678T412	Vitamin b12
M169T48_2	Norharmane	M388T390	Thifensulfuron-methyl
M177T135_2	Serotonin	M721T400	2-thiophenecarboxamide, 3-[(aminocarbonyl)amino]-5-[4-(4-morpholinylmethyl)phenyl]-
M180T107	Adrenochrome	M127T188	Melamine
M190T338	1h-indole-2-carboxylic acid, ethyl ester	M142T57	N-tert-butyl-6-methoxy-1,3,5-triazine-2,4-diamine
M186T200	Indolelactic acid	M216T183	Atrazine
M190T181	Methyl indole-3-acetate	M367T154	Cyhexatin
M190T280	N-Acetyl-DL-methionine	M499T422	Silydianin
M629T245	Octadecamethyloctasiloxane	M501T353	Isorhamnetin 3-galactoside
M131T153	Cinnamic acid ethyl ester	M509T228	Syringetin 3-galactoside
M147T50	Trans-2-hydroxycinnamic acid	M519T247_2	3,5-dihydroxy-2-(4-hydroxyphenyl)-6-(3-methylbut-2-enyl)-7-[3,4,5-trihydroxy-6-(hydroxymethyl)oxan-2-yl]oxy-2,3-dihydrochromen-4-one
M149T210	Trans-cinnamic acid	M521T211	Anthocyanidin base+3o, o-malonylhex
M547T267	5,7-dihydroxy-2-phenyl-6-[3,4,5-trihydroxy-6-(hydroxymethyl)oxan-2-yl]-8-(3,4,5-trihydroxyoxan-2-yl)chromen-4-one	M535T301	Flavone base + 3o, c-pen, c-pen
M177T238	Trans-ferulic acid	M163T272	P-coumaric acid
M181T36	Phenanthroline	M563T328	Schaftoside
M196T276	.alpha.-methyl-l-tyrosine	M565T329	Flavone base + 3o, c-hex, c-pen
M207T51	P-methoxycinnamic acid ethyl ester	M569T446_1	3-[5,7-dihydroxy-2-(4-methoxyphenyl)-4-oxo-2,3-dihydrochromen-3-yl]-5,7-dihydroxy-2-(4-methoxyphenyl)-2,3-dihydrochromen-4-one
M221T77	Benzyl cinnamate	M577T297	Rhoifolin
M483T259	[(2r,3s,4s,5r,6r)-3,4,5-trihydroxy-6-[2-(3-hydroxy-5-oxooxolan-3-yl)propoxy]oxan-2-yl]methyl (e)-3-(3,4-dihydroxyphenyl)prop-2-enoate	M577T398	Saponarin
M239T180	3,4,5-trimethoxycinnamic acid		
M328T180	1-o-trans-cinnamoyl-beta-d-glucopyranose	M581T28	Sciadopitysin
M339T401_1	4-demethylsimmondsin 2'-ferulate	M581T376	Naringin dihydrochalcone
M344T349	Cinnamyl 3,4-dihydroxy-.alpha.-cyanocinnamate	M593T346	Lonicerin
M383T185_1	Rosmarinic acid	M597T384	Eriocitrin
M387T52	1-o-b-d-glucopyranosyl sinapate	M603T329	Leucoside

M469T207	G(8-o-4)fa sulfate	M609T338	Rutin
M517T238	[6-[3,4-dihydroxy-2,5-bis(hydroxymethyl)oxolan-2-yl]oxy-3,4,5-trihydroxyoxan-2-yl]methyl (e)-3-(4-hydroxy-3-methoxyphenyl)prop-2-enoate	M225T40	3,5-dimethoxy-4-hydroxycinnamic acid
M517T480	Sibiricose a5	M609T266	Spinosine
M639T418	Plantamajoside	M615T304	Fortunellin
M647T276	Acteoside	M617T328_2	Poncirin
M684T496_3	6-acetylacteoside	M617T390	Kaempferol-3-glucoside-3"-rhamnoside
M745T290	Scropolioside d	M619T384	Eriodictyol 7-o-neohesperidoside
M115T201	Psoralen	M621T476	4'-o-(glca(1-2)glca) apigenin
M147T38	Coumarin	M623T233_1	Isorhamnetin-3-o-rutinoside
M161T116	7-hydroxycoumarin	M625T414	Isorhamnetin 3-neohesperidoside
M177T211	7-hydroxy-4-methylchromen-2-one	M625T480	Peonidin-3,5-o-di-beta-glucoside
M193T49	Scopoletin	M631T346_1	Diosmin
M221T28	7-hydroxycoumarin-4-acetic acid	M645T245	Pectolinarin
M237T396	Gly-Thr	M677T259	Icariin
M342T189	Skimmin	M699T348	Theaflavin 3-gallate
M757T288	3-[(2s,3r,4s,5r,6r)-4-[(2s,3r,4s,5s,6r)-4,5-dihydroxy-6-(hydroxymethyl)-3-[(2s,3r,4s,5r)-3,4,5-trihydroxyoxan-2-yl]oxyoxan-2-yl]oxy-3,5-dihydroxy-6-(hydroxymethyl)oxan-2-yl]oxy-2-(3,4-dihydroxyphenyl)-5,7-dihydroxychromen-4-one	M725T415	Camelliaside b
M361T351	4-methylumbelliferyl .beta.-d-glucopyranoside	M749T407	5-hydroxy-3-[(2s,3r,4r,5s)-3-hydroxy-5-(hydroxymethyl)-4-[(2s,3r,4s,5s,6r)-3,4,5-trihydroxy-6-(hydroxymethyl)oxan-2-yl]oxyoxolan-2-yl]oxy-2-(4-hydroxyphenyl)-7-[(2s,3r,4r,5r,6s)-3,4,5-trihydroxy-6-methyloxan-2-yl]oxychromen-4-one
		M515T349	Isopimpinellin
M404T444	Pteryxin	M351T394	4-methylumbelliferyl .beta.-d-glucuronide
		M763T328	Robinin
M405T112	Bergaptol	M769T504	4'-o-(2'-e-coumaroyl glua)(1-2)glua) apigenin
M429T130	Isomajdine	M1051T464	Cyanidin 3-o-[2"-o-(xylosyl)-6"-o-(p-o-(glucosyl)-p-coumaroyl) glucoside] 5-o-glucoside
M455T332_1	[3-hydroxy-1-(4-methoxy-7-oxofuro[3,2-g]chromen-9-yl)oxy-3-methylbutan-2-yl] (e)-2-methylbut-2-enoate	M779T409	5,7-dihydroxy-2-[4-[(2s,3r,4s,5s,6r)-3,4,5-trihydroxy-6-(hydroxymethyl)oxan-2-yl]oxyphenyl]-3-[(2s,3r,4s,5s,6r)-3,4,5-trihydroxy-6-[[(2r,3r,4r,5r,6s)-3,4,5-trihydroxy-6-methyloxan-2-yl]oxymethyl]oxan-2-yl]oxychromen-4-one
M753T326	3-[4,5-dihydroxy-3-[(2r,3r,4r,5r,6s)-3,4,5-trihydroxy-6-methyloxan-2-yl]oxy-6-[[(2r,3r,4r,5r,6s)-3,4,5-trihydroxy-6-methyloxan-2-yl]oxymethyl]oxan-2-yl]oxy-5-hydroxy-2-(4-hyd	M793T395	3-[(2s,3r,4s,5s,6r)-4,5-dihydroxy-3-[(2r,3r,4r,5r,6s)-3,4,5-trihydroxy-6-methyloxan-2-yl]oxy-6-[[(2r,3r,4r,5r,6s)-3,4,5-trihydroxy-6-methyloxan-2-yl]oxymethyl]oxan-2-yl]oxy-5,7-dihydroxy-2-(4-hyd

	roxyphenyl)-7-methoxychromen-4-one		roxyphenyl)-6-methoxychromen-4-one
M797T469	[3,4-dihydroxy-6-[5-hydroxy-2-(4-hydroxyphenyl)-4-oxo-7-[3,4,5-trihydroxy-6-(hydroxymethyl)oxan-2-yl]oxychromen-3-yl]oxy-5-[(2s,3r,4r,5r,6s)-3,4,5-trihydroxy-6-methyloxan-2-yl]oxyoxan-2-yl]methyl acetate	M573T289	(-)-oxypeucedanin
M811T492	Haploperoside c acetate	M809T265	Xanthorhamnin
M147T221_2	Bisdemethoxycurcumin	M817T23	Flavonol base + 4o, 1meo, o-hex-hex, o-hex
M217T210	Demethoxycurcumin	M827T265	2-[[4-[4,6-dihydroxy-3-(3-methylbut-2-enyl)-2-[3,4,5-trihydroxy-6-(hydroxymethyl)oxan-2-yl]oxyphenyl]-3-hydroxy-2-(4-hydroxyphenyl)-5-methoxy-3,4-dihydro-2h-chromen-7-yl]oxy]-6-(hydroxymethyl)oxane-3,4,5-triol
M251T205	Bisphenol m	M857T420	Epimedin c
M335T436	(2s,3s)-3,5,7-trihydroxy-6-methyl-2-(3,4,5-trihydroxyphenyl)-2,3-dihydrochromen-4-one	M861T302_2	Epimedin a
M417T298	Daidzin	M863T474	Apigenin 7-glucoside
M369T227_1	Curcumin	M645T466	[(2r,3s,4s,5r,6r)-6-[1,7-bis(4-hydroxyphenyl)-5-oxoheptan-3-yl]oxy-3,4,5-trihydroxyoxan-2-yl]methyl (e)-3-(4-hydroxyphenyl)prop-2-enoate
M511T329	Bromadiolone	M771T393	Flavonol base + 4o, o-hex-dhex, o-hex
M517T220	Rottlerin	M901T335	[(2s,3r,4s,5s,6r)-2-[3-[(2s,3r,4s,5s,6r)-4,5-dihydroxy-6-(hydroxymethyl)-3-[(2s,3r,4r,5r,6s)-3,4,5-trihydroxy-6-methyloxan-2-yl]oxyoxan-2-yl]oxy-5-hydroxy-2-(4-hydroxyphenyl)-4-oxochromen-7-yl]oxy-4,5-dihydroxy-6-(hydroxymethyl)oxan-3-yl] (e)-3-(4-hydroxyphenyl)prop-2-enoate
M617T249	(2r,3s,4s,5r,6r)-6-[1,7-bis(4-hydroxyphenyl)heptan-3-yloxy]-5-[(2s,3r,4r)-3,4-dihydroxy-4-(hydroxymethyl)oxolan-2-yl]oxy-2-(hydroxymethyl)oxane-3,4-diol	M241T189	Rotenone
M865T535	Procyanidin c1	M243T239	Equol
M917T230	3-[(2s,3r,4s,5r,6r)-3-[(2s,3r,4r,5s,6s)-3,5-dihydroxy-6-methyl-4-[(2s,3r,4s,5s,6r)-3,4,5-trihydroxy-6-(hydroxymethyl)oxan-2-yl]oxyoxan-2-yl]oxy-4,5-dihydroxy-6-[[(2r,3r,4r,5r,6s)-3,4,5-trihydroxy-6-methyloxan-2-yl]oxymethyl]oxan-2-yl]oxy-2-(3,4-dihydroxyphenyl)-5,7-dihydroxychromen-4-one	M285T39	Maackiaine
M1127T328_2	APIIN	M333T24	Daidzein 4'-sulfate
M223T189	Flavone	M337T37_1	Psoralidin
M253T209	4'-methoxyflavanone	M407T411	6a,12a-didehydroamorphigenin
M269T36	Fustin	M445T110	Trifolirhizin

M289T13	(+)-catechin	M463T374	Tectoridin
M323T184_2	Hesperetin	M491T242	3',4',6-trimethoxyisoflavone-7-o-.beta.-d-glucopyranoside
M357T51_1	3-hydroxy-7,8,2',3'-tetramethoxyflavone	M499T457	Mundulone acetate
M360T55	3',4',5,7-tetrahydroxy-3,6,8-trimethoxyflavone	M533T212	6"-o-malonylglycitin
M367T373	Isoanhydroicaritin		
M373T41	5,6,7,3',4'-pentamethoxyflavone	M545T399	Iridin
M375T99	7-o-benzylluteolin		
M391T268	Glabrol	M659T341	9-methoxy-7-[4-[3,4,5-trihydroxy-6-[[3,4,5-trihydroxy-6-(hydroxymethyl)oxan-2-yl]oxymethyl]oxan-2-yl]oxyphenyl]-[1,3]dioxolo[4,5-g]chromen-8-one
M677T439_1	6-hydroxy-3-[3-hydroxy-4-[3,4,5-trihydroxy-6-[[3,4,5-trihydroxy-6-(hydroxymethyl)oxan-2-yl]oxymethyl]oxan-2-yl]oxyphenyl]-5,7-dimethoxychromen-4-one	M395T407	Tangeritin
M419T300_3	Isoliquiritin	M237T37	Butein
M425T284	Nobiletin	M281T184	2'-hydroxy-6'-methyl-3,4-methylenedioxychalcone
M433T116	Prunin	M299T135_1	2,4-dimethoxy-2'-hydroxy-5'-methylchalcone
M433T204	Isovitexin	M327T244	4'-hydroxy-2'-methyl-3,4,5-trimethoxychalcone
M435T329_2	Naringenin-7-o-glucoside	M329T33	4,2'-dihydroxy-3,4',6'-trimethoxychalcone
M443T263	(-)-catechin gallate	M375T298	2'-hydroxy-2,4,4',5,6'-pentamethoxychalcone
M445T296	Swertisin	M435T180	1-[2,4-dihydroxy-3-[(2s,3r,4r,5s,6r)-3,4,5-trihydroxy-6-(hydroxymethyl)oxan-2-yl]phenyl]-2-hydroxy-3-(4-hydroxyphenyl)propan-1-one
M445T36_1	(z)-2,6-dimethyl-7-(4-methyl-5-oxooxolan-2-yl)-3-[[3,4,5-trihydroxy-6-(hydroxymethyl)oxan-2-yl]oxymethyl]hept-5-enoic acid	M165T117	Phenyllactic acid
M449T169_2	Isosakuranin	M212T237	Methyldopa
M449T234	Luteolin 7-glucoside	M451T88	Carbidopa
M451T218	Astilbin	M135T435	Resveratrol
M453T259	Silychristin	M391T187_2	Polydatin
M453T299	Liquiritin	M407T281	Mulberroside a
M453T51_1	Silybin	M421T216	Rhaponticin
M459T233	Phlorizin	M449T356	Deoxyrhapontin
M461T158	Homoplantaginin	M329T333	Benzoic acid + 1o, 1meo, o-hex
M463T261	Quercetin 3-glucoside	M333T206	Picroside ii
M463T290	Hyperoside	M359T328	Benzoic acid + 1o, 2meo, o-hex
M465T215_2	Neohesperidin	M559T302	Gomisin c
M473T329_2	Marein	M713T328	Methyl (5z)-5-[2-(4-hydroxy-3-methoxybenzoyl)oxyethylidene]-4-[2-[2-(4-hydroxyphenyl)ethoxy]-2-oxoethyl]-6-[(2s,3r,4s,5s,6r)-3,4,5-trihydroxy-6

			-(hydroxymethyl)oxan-2-yl]oxy-4h-pyran-3-carboxylate
M463T260_2	[(4r,5s,6r,6as,7r,10ar,11br)-5-acetyloxy-6-hydroxy-10a-methoxy-4,7,11b-trimethyl-9-oxo-1,2,3,4a,5,6,6a,7,11,11a-decahydronaphtho[2,1-f][1]benzofuran-4-yl]methyl acetate	M444T177	Borrelidin
M501T323	Flavine mononucleotide	M447T204_2	Quercitrin
M125T376	3R-hydroxy-butanoic acid	M445T370	Tetracycline
M130T134	Paxilline	M461T392_1	Kotanin
M133T194	(2s,3s)-(-)-3-phenylglycidol	M477T353	Isorhamnetin-3-o-glucoside
M144T288	DL-2-Aminoadipic acid	M463T301	Peonidin 3-galactoside cation
M151T181	4-Acetoxyphenol	M473T374	Sofalcone
M162T327	L-Erythro-4-Hydroxyglutamic acid	M485T29	Kendomycin
M167T130	Bendiocarb	M487T277	Biotin
M177T377	2-dehydro-3-deoxy-d-gluconate	M487T458	Oxyresveratrol
M182T378	L-Tyrosine	M493T426	3beta-hydroxydeoxodihydrodeoxygedunin
M188T303_1	DL-Indole-3-lactic acid	M495T157	1-(10z-heptadecenoyl)-sn-glycero-3-phospho-(1'-rac-glycerol)
M195T150	Hydroferulic acid	M207T240	6,7-dimethylesculetin
M205T67	5h-dibenzo[a,d]cyclohepten-5,10-imine, 10, 11-dihydro-5-methyl-, (5s,10r)-	M506T591	Calcimycin
M207T73	D-Arabinono-1,4-lactone	M507T347	Metaflumizone
M213T156	Perillyl alcohol	M532T331_1	1- piperidinecarboxylic acid, 4-[bis(1,3-benzodioxol-5-yl)hydroxymethyl]-, 2,2,2-trifluoro-1-(trifluoromethyl)ethyl ester
M233T141	Pyroxamide	M537T38	2-mohpda [dmed-fahfa]
M219T231	L-abrine	M565T419	Ginkgetin
M526T387	Epirubicin	M573T136	Oligomycin a
M235T123	5,6,7-trimethoxycoumarin		
M270T200	Oxotremorine	M573T39_2	2-sdahma [dmed-fahfa]
M272T223	Normorphine	M577T466	Gossypol
M282T38_1	10-hydroxymorphine	M599T528	4-[3,4,5-trihydroxy-6-(hydroxymethyl)oxan-2-yl]oxy-9-(3,4,5-trimethoxyphenyl)-5a,6,8a,9-tetrahydro-5h-[2]benzofuro[6,5-f][1,3]benzodioxol-8-one
M283T55	Brazilein	M285T426	His-Glu
M601T487	(3s,4s)-5-[(3s,4s)-4,10-dihydroxy-7,9-dimethoxy-3-methyl-3,4-dihydro-1h-benzo[g]isochromen-5-yl]-7,9-dimethoxy-3-methyl-3,4-dihydro-1h-benzo[g]isochromene-4,10-diol	M602T281	Montelukast sulfoxide
M291T429_2	Pro-Asp	M607T204_2	Terragine e
M306T285	Arg-Met	M619T394	Phosphonic acid, p-[2-[3-[[(1-benzoyl-4-piperidinyl)methylamino]carbonyl]-2-naphthalenyl]-1-(1-naphthalenyl)-2-oxoethyl]-
M315T217	Picrotoxinin	M662T434	.beta.-nicotinamide adenine dinucleotide(NAD)

M315T28	Rofecoxib	M673T49	1-Palmitoyl-2-oleoyl-sn-glycero-3-phosphate
M331T154	Nornotatic acid	M68T311	Pyrrol
M621T406	Jacaglabroside b	M698T180	2-tpahhda [dmed-fahfa]
M336T69_1	Isopentenyladenosine	M709T305	Specnuezhenide
		M709T420	Secoisolariciresinol diglucoside
M337T491	Arg-Tyr	M734T147_2	PS(16:0/16:0)
M341T132_1	1-Stearoyl-rac-glycerol	M744T210_1	Montelukast acyl-.beta.-d-glucuronide
M343T445	4-O-.beta.-Galactopyranosyl-D-mannopyranose	M755T409	Cyanidin 3-(2g-glucosylrutinoside)
M352T141	Trans-zeatin-riboside		
M353T27	Rauwolscine		
M357T44	Pioglitazone	M759T118	Thioetheramide-PC
M364T212	Thymidine 5'-monophosphate	M765T262	Eleutheroside e
M369T382	Digalacturonic acid	M787T210	Oligomycin b
M379T258	Daunorubicin	M791T376	Calphostin c
M383T26_1	Isorhynchophylline	M80T64	Pyridine
M397T27_2	Mitragynine	M842T66	Pg 42:10
M397T381	Phenacetine	M903T292	[3,4-dihydroxy-5-[2-methylidene-4-[(2r,3r,4s,5s,6r)-3,4,5-trihydroxy-6-[[2-methylidene-4-[(2r,3r,4s,5s,6r)-3,4,5-trihydroxy-6-[(4-hydroxy-2-methylidenebutanoyl)oxymethyl]oxan-2-yl]oxybutanoyl]oxymethyl]oxan-2-yl]oxybutanoyl]oxyoxan-2-yl]methyl 4-hydroxy-2-methylidenebutanoate
M403T450	Maltitol	M407T151	Ginkgolide b
M405T233	Furathiocarb	M961T264	Man9
M927T170	(2r,3r,4s,5s,6r)-2-[[(2r,3s,4s,5r,6s)-6-[4-[3-[3,5-dimethoxy-4-[(2s,3r,4s,5s,6r)-3,4,5-trihydroxy-6-(hydroxymethyl)oxan-2-yl]oxyphenyl]-1,3,3a,4,6,6a-hexahydrofuro[3,4-c]furan-6-yl]-2,6-dimethoxyphenoxy]-3,4,5-trihydroxyoxan-2-yl]methoxy]-6-(hydroxymethyl)oxane-3,4,5-triol	M415T39	Ascorbic acid 6-palmitate
		M419T281	Aloin A
		M425T261_2	5a,6-anhydrotetracycline
