# Supplementary figures and images for: The Impact of Growth Years on the Medicinal Material Characteristics and Metabolites of Stellaria dichotoma L. var. lanceolata Bge. Reveals the Optimal Harvest Age
Source: Plants (Basel). 2023 Jun 12;12(12):2286. doi: 10.3390/plants12122286 (PMC10304078; doi:10.3390/plants12122286)

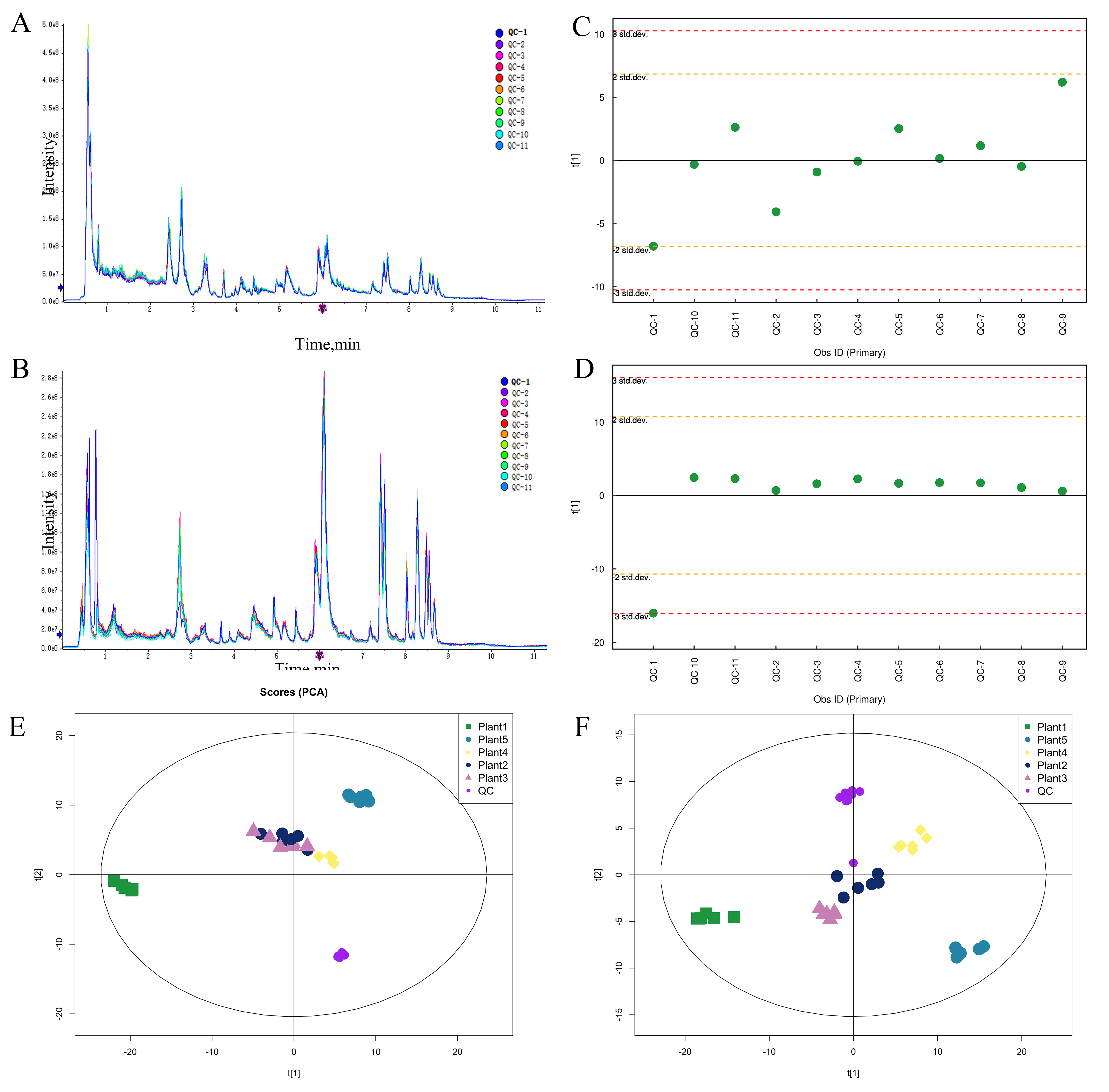

Supplement: Supplementary file 1 [file plants-12-02286-s001.zip › Supplementary Figure S1.jpg]

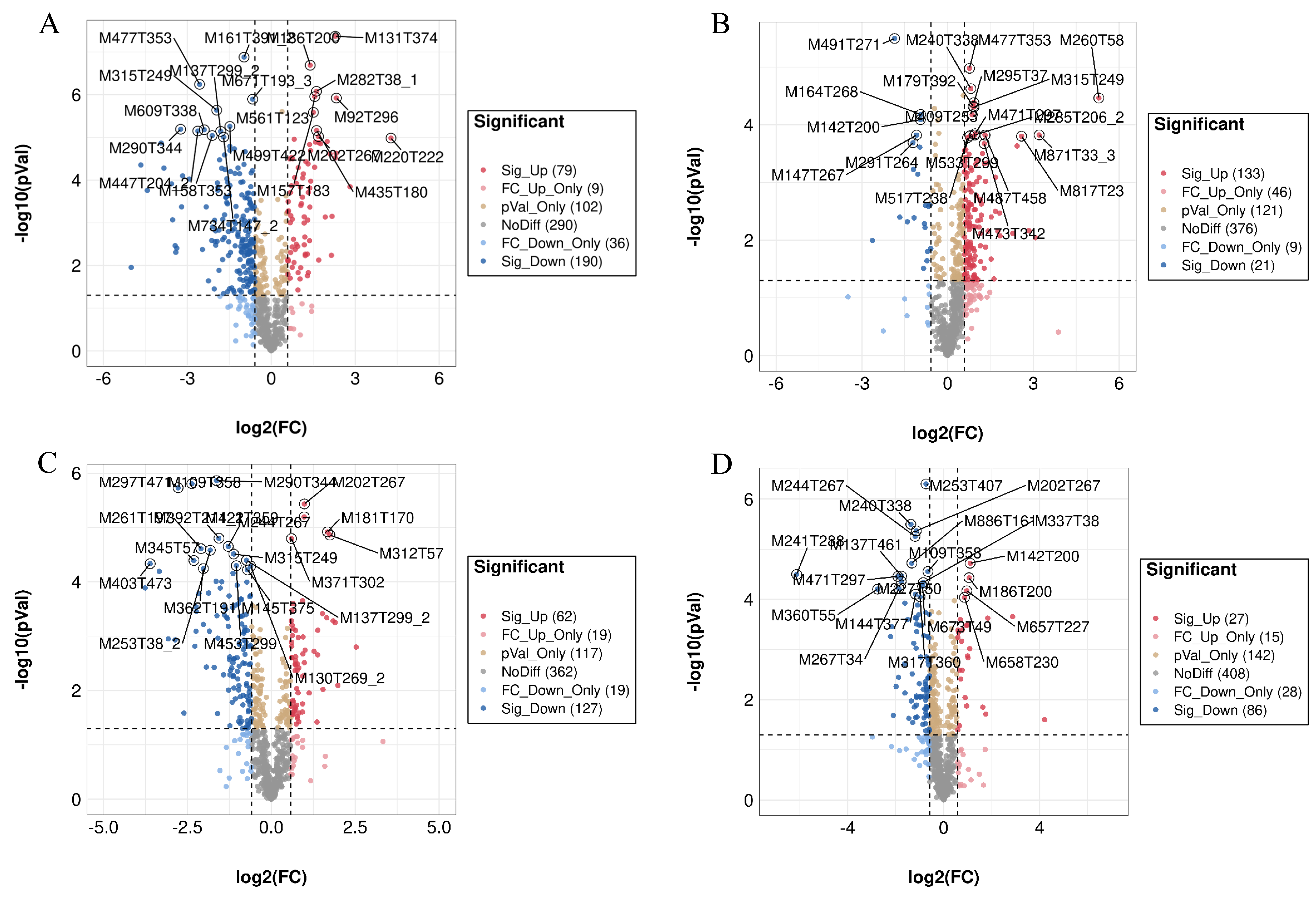

Supplement: Supplementary file 1 [file plants-12-02286-s001.zip › Supplementary Figure S2.jpg]

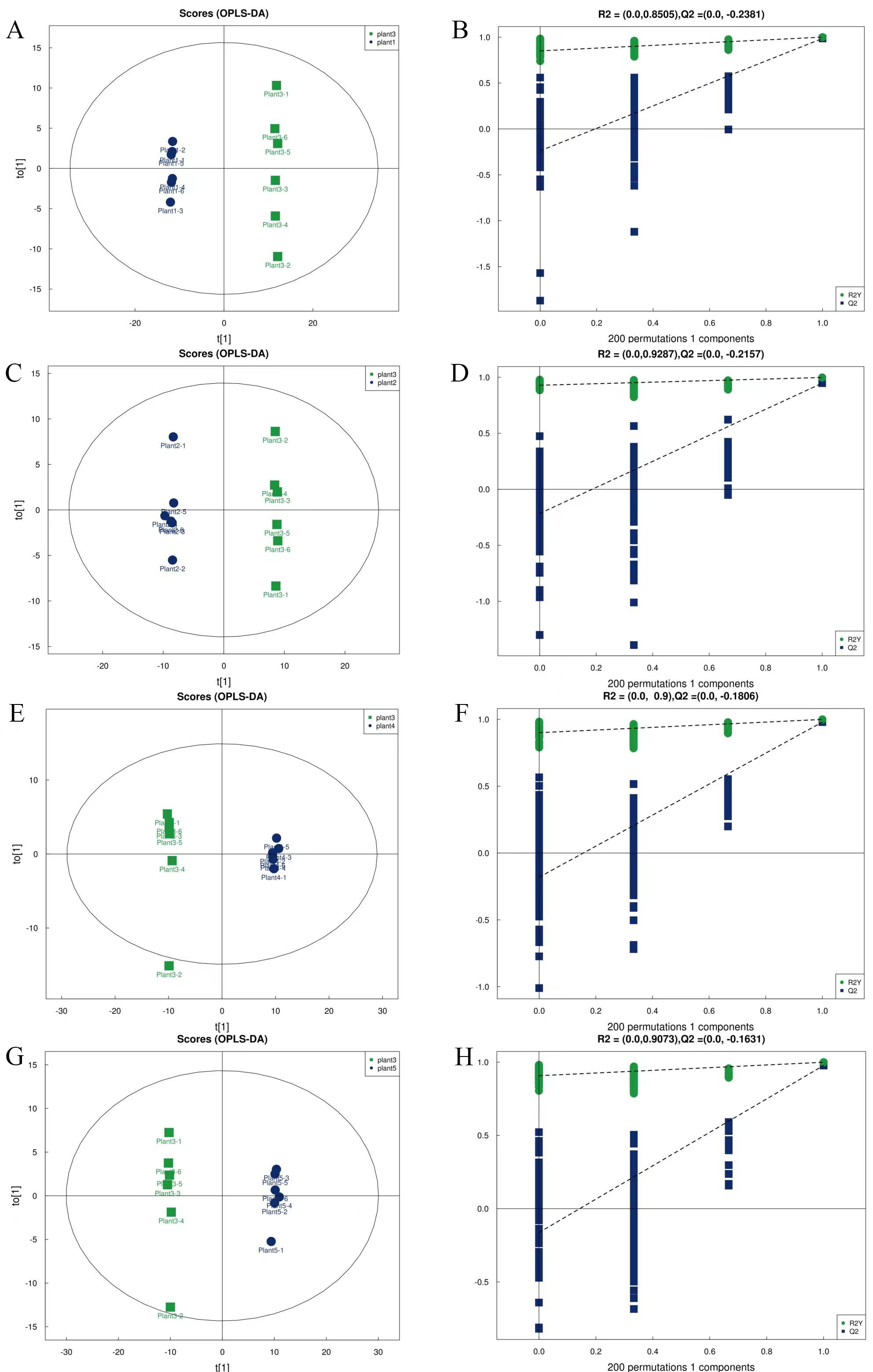

Supplement: Supplementary file 1 [file plants-12-02286-s001.zip › Supplementary Figure S3.jpg]

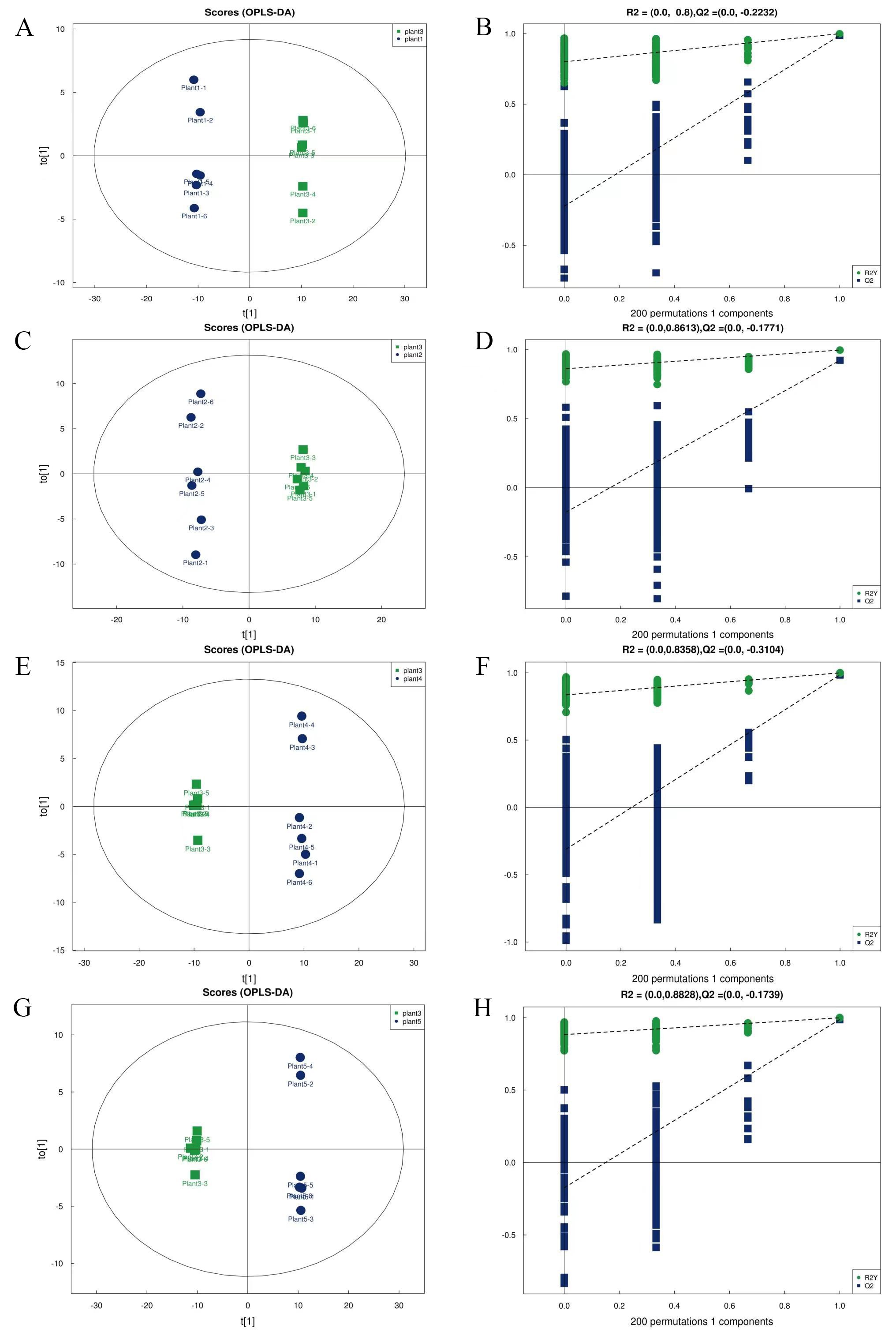

Supplement: Supplementary file 1 [file plants-12-02286-s001.zip › Supplementary Figure S4.jpg]

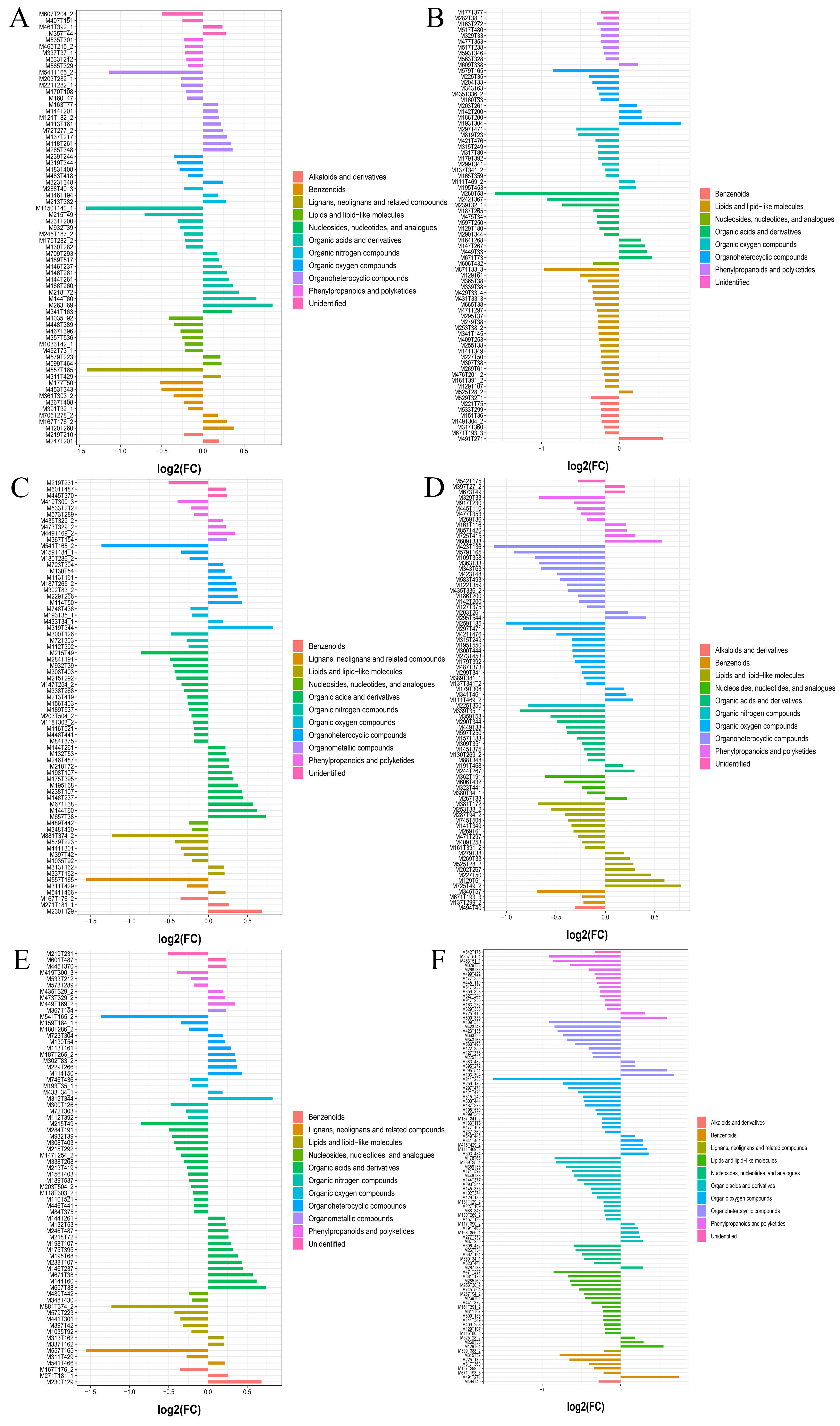

Supplement: Supplementary file 1 [file plants-12-02286-s001.zip › Supplementary Figure S5.jpg]
